# Constitutive activation of canonical Wnt signaling disrupts choroid plexus epithelial fate

**DOI:** 10.1038/s41467-021-27602-z

**Published:** 2022-02-02

**Authors:** Arpan Parichha, Varun Suresh, Mallika Chatterjee, Aditya Kshirsagar, Lihi Ben-Reuven, Tsviya Olender, M. Mark Taketo, Velena Radosevic, Mihaela Bobic-Rasonja, Sara Trnski, Michael J. Holtzman, Nataša Jovanov-Milosevic, Orly Reiner, Shubha Tole

**Affiliations:** 1 Department of Biological Sciences, Tata Institute of Fundamental Research, Mumbai, 400005 India; 2 Department of Molecular Genetics, Weizmann Institute of Science, Rehovot, Israel; 3 Division of Experimental Therapeutics Graduate School of Medicine, Kyoto University (Yoshida-Konoé-Cho, Sakyo), Kyoto, 606-8501 Japan; 4 Croatian Institute for Brain Research, Department of Medical Biology, School of Medicine University of Zagreb, Šalata 12, Zagreb, Croatia; 5 University Hospital Centre Zagreb, Department of Gynecology and Department of Pathology and Cytology, Petrova 13, Zagreb, Croatia; 6 Pulmonary and Critical Care Medicine, Washington University, St. Louis, 63110 MO US; 7 Present Address: Amity Institute of Neuropsychology and Neurosciences, Amity University, Noida, India

**Keywords:** Differentiation, Embryonic induction

## Abstract

The choroid plexus secretes cerebrospinal fluid and is critical for the development and function of the brain. In the telencephalon, the choroid plexus epithelium arises from the *Wnt*- expressing cortical hem. Canonical Wnt signaling pathway molecules such as nuclear β-CATENIN are expressed in the mouse and human embryonic choroid plexus epithelium indicating that this pathway is active. Point mutations in human *β-CATENIN* are known to result in the constitutive activation of canonical Wnt signaling. In a mouse model that recapitulates this perturbation, we report a loss of choroid plexus epithelial identity and an apparent transformation of this tissue to a neuronal identity. Aspects of this phenomenon are recapitulated in human embryonic stem cell derived organoids. The choroid plexus is also disrupted when *β-Catenin* is conditionally inactivated. Together, our results indicate that canonical Wnt signaling is required in a precise and regulated manner for normal choroid plexus development in the mammalian brain.

## Abstract (web summary)

The cerebrospinal fluid-secreting choroid plexus needs a balanced level of canonical Wnt signaling. Here the authors show that if this signaling is over-activated, the choroid plexus loses its properties and function, and transforms to a neuronal identity.

## Introduction

The Choroid plexus (ChP) produces cerebrospinal fluid (CSF) which plays an important role in the developing and adult brain. The CSF provides protective functions, such as cushioning against mechanical damage, nutritive function, and signaling functions, providing molecular cues for the developing brain^1,2^. The ChP separates the blood from the CSF creating a checkpoint for regulating the entry and exit of factors^2,3^. The ChP is also the gateway for immune cells entering or exiting the brain^4^.

The ChP epithelium (ChPe) arises from adjacent neuroepithelial tissue in the telencephalon, diencephalon, and hindbrain. In the telencephalon, the non-neuronal ChPe and a sub-population of Cajal-Retzius (CR) neurons both arise from the cortical hem^5–7^, a midline telencephalic signaling center enriched in genes that belong to the BMP and WNT protein families. The telencephalic hem has been identified in several vertebrate classes^8^. In the opossum *Monodelphis domestica*, BrdU positive cells arise from the hem and populate the growing telencephalic ChPe in a “conveyor belt” like manner^9^. Consistent with this model, when the size of the cortical hem increases^10^, decreases^11^ or is missing altogether^12^, there is a parallel change in the extent of the ChP.

The mechanisms that regulate the specification of the ChPe are important from developmental, disease, and evolutionary perspectives. Obvious candidates for the control of ChPe development are the signaling molecules expressed in the neuroepithelial progenitor domains. The hem expresses several members of the Bmp and Wnt families^12,13^. When the expression of these signaling molecules in the hem is compromised, such as in the *Gli3* null (Xt^J^) mutant, neither the ChP nor the hippocampus is specified normally^12,14^. Bmp signaling is critical for ChPe specification. The ChP is missing in the *BmpR1a* null mutant mouse^15,16^, and *Bmp4* is sufficient for ChPe induction in vitro^16,17^. The role of hem-derived Wnt ligands in ChPe development appears to be equally important, though not well explored. *Wnt5a*, expressed in the hem, appears to be required for maintaining the size and the cytoarchitecture of the ChPe^18^. A role for canonical Wnt signaling in ChPe development is suggested by the observation that the choroid plexus appears to be reduced in the *Wnt3a* null mutant^19^. Furthermore, the addition of WNT3A augments the ChPe-inducing ability of BMP4 in a three-dimensional aggregation culture derived from embryonic stem cells (ESCs)^17^.

In this study, we demonstrate that molecules downstream of canonical Wnt signaling, such as nuclear β-CATENIN (CTNNB1), are present in vivo at embryonic stages in the human and mouse ChPe. Mutations in particular domains of human *β-CATENIN* have been reported to cause constitutive activation of this pathway since they interfere with β-CATENIN phosphorylation and consequent degradation^20^. Specifically, exon 3 of the *β-CATENIN* gene is a hotspot for several mutations, including point mutations and deletions associated with various forms of cancer^21^. The effects of these mutations on ChP-related disorders have not been studied, even though ChP papillomas have been reported to display upregulation of canonical Wnt signaling^22^. Therefore, we focused our studies on examining the effects of constitutive activation of β-CATENIN in the mouse ChPe using a strategy in which exon 3 can be conditionally deleted. We discovered that this perturbation causes a loss of ChPe identity and, surprisingly, a gain of neuronal identity, together with comprehensive transcriptomic changes that are consistent with the activation of canonical Wnt signaling. We paralleled these studies in a human ESC (hESC)- derived organoid model in which treatment with a low level of a canonical Wnt agonist together with BMP4 resulted in a ChPe-like fate, but over-activation with high levels of the Wnt agonist recapitulated the loss of ChPe identity seen in the mouse. Loss of *β-catenin* in the mouse resulting in an absence of canonical Wnt signaling^23^ also disrupted ChPe development.

Together, our results indicate that controlled levels of canonical Wnt signaling is critical for ChPe specification and morphogenesis and that this mechanism appears to be conserved in humans and rodents.

## Results

### The human fetal ChPe and the embryonic mouse ChPe display components of canonical Wnt signaling

**Fig. 1:**
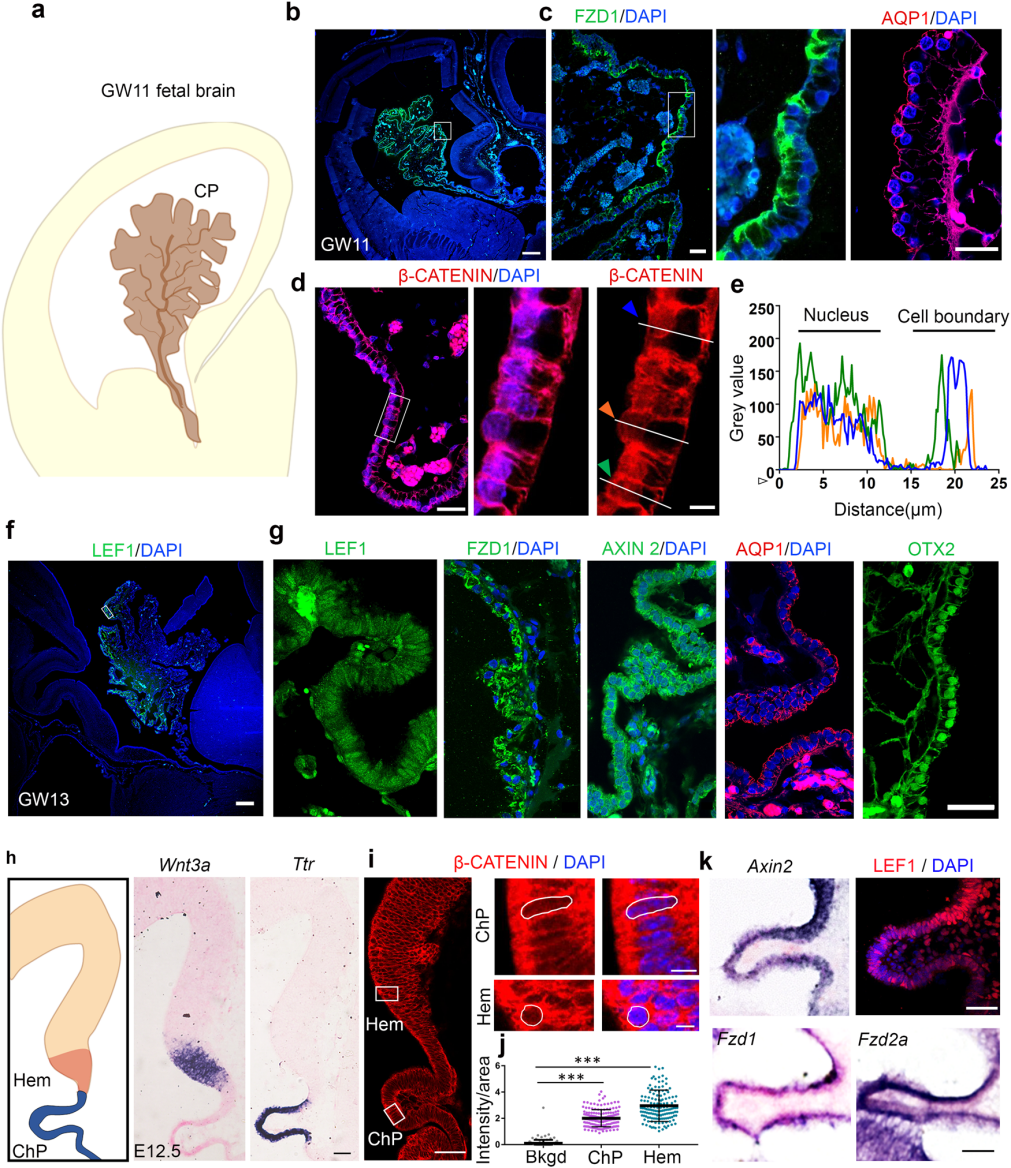
Canonical Wnt signaling components are present in the developing human and mouse choroid plexus epithelium. **a**–**g** Canonical Wnt signaling pathway molecules FZD1, β-CATENIN, LEF1, and AXIN2, and also ChPe markers OTX2 and AQP1 are detected at gestational week (GW) 11 (**b**–**e**) and GW13 (**f**, **g**) in the human choroid plexus epithelium. **d**, **e** β-CATENIN immunohistochemistry intensity measurements across three ChPe cells (identified by colored arrowheads in d corresponding to traces in **e**). β-CATENIN intensity is seen in the nucleus, indicative of active canonical Wnt signaling and the boundaries between cells. **b**–**g** Representative images of sections taken from 1 fetal brain at each age. **h** In the E12.5 mouse telencephalon, *Wnt3a* and *Ttr* expression identifies the hem the ChPe, respectively. **i**, **j** Nuclear localization of β-CATENIN and its quantitation (Bkgd, background; scatterplot represents mean ± SEM; *n* = 150 nuclei; *N* = 3 brains (biologically independent replicates) examined over 3 independent experiments. **k** Canonical Wnt signaling components *Axin2, Fzd1, Fzd2a*, and LEF1 are expressed in the E12.5 mouse ChPe. (**h**, **k**) Representative images of sections taken from *N* = 7 brains (biologically independent replicates) examined in 5 independent experiments. Statistical test (**j**): One Way ANOVA followed by post hoc Dunnett’s multiple comparison test (*p* < 0.0001), *p < 0.05, **p < 0.01, ***p < 0.001, ns if *p* value > 0.05. Scale bars: 500 μm (all panels in **b** and **f**); 50 μm (all panels in **d**, **i** low mag, **g**, **h**, and **k**); 10 μm (**d** and **i** high mag). Boxed regions (**b**, **c**, **d**, **f**, **i**) are shown at high magnification in the adjacent panels. Further information on replicates and reproducibility for this figure is mentioned in the “Statistics and Reproducibility” section of the Methods. Source data are provided as a Source Data file.

We examined whether the molecular machinery required for processing canonical Wnt signaling is present in the developing ChPe at the relevant stages in both human and mouse embryos. In humans, the telencephalic ChP develops between gestational weeks 8 to 12. We collected human fetal samples from GW11 and GW13 and performed immunohistochemical analysis for Wnt signaling components. The human ChP is identified by its fan-like morphology within the telencephalic ventricles (Fig. 1a, b), and the presence of AQP1 and OTX2 that are established ChPe markers (Fig. 1a–g). The canonical Wnt receptor, FZD1, is detected throughout the ChPe (Fig. 1b). LEF1, AXIN2, and β-CATENIN are also present, consistent with canonical Wnt signaling. In particular, β-CATENIN was detected in the nucleus, (Fig. 1d, e) indicating that canonical Wnt signaling is likely to be active in the human ChPe at early stages during ChP development.

In the embryonic day E12.5 mouse, the hem and the ChPe are identified by the expression of *Wnt3a* and the thyroid hormone T4 carrier transthyretin (*Ttr*), respectively (Fig. 1h). The expression of several canonical Wnt signaling pathway molecules was detected in the ChPe including receptors such as *Frizzled 1* and *2a* (*Fzd1, Fzd2a*), a target gene *Axin2*, and the transcriptional regulators β-CATENIN and LEF1 were present in nuclei of ChPe cells (Fig. 1i–k). These data suggest that the embryonic human and mouse ChPe have the capability of processing canonical Wnt signaling.

### Disrupting β-CATENIN function in the hem and ChPe

**Fig. 2:**
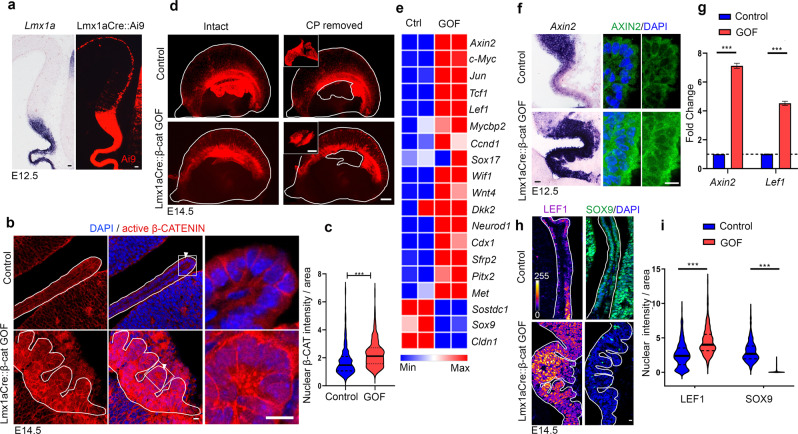
Stabilization of β-CATENIN leads to activation of canonical Wnt signaling. (**a**) At E12.5, in situ hybridization for *Lmx1a* shows endogenous expression; the Ai9 reporter labels the *Lmx1a*-expressing lineage, including the hem, ChPe, and hem-derived Cajal-Retzius cells. (**b**, **c**) At E14.5, nuclear localization of active (non-phosphorylated) β-CATENIN is increased in the Lmx1aCre::β-cat GOF ChPe compared with controls (arrowheads) as seen by (**b**) immunohistochemistry and (**c**) a violin plot representing quantitation of *n* = 294 nuclei (control) and *n* = 301 (GOF); *N* = 3 brains (biologically independent replicates) for each genotype examined over 2 separate experiments. Boxed regions (**b**) are shown at high magnification in the adjacent panels. (**d**) Microdissection of E14.5 Ai9-labeled telencephalic hemispheres to isolate ChP (inset) for RNA extraction. (**a**, **d**) Representative images of sections taken from *N* = 3 brains (biologically independent replicates) examined over 2 independent experiments. (**e**) Heatmap (showing normalized reads) of Wnt signaling target genes from RNA-seq data from control and Lmx1aCre::β-Catenin GOF ChP (*N* = 2 biologically independent replicates). Color key: red (high expression) and blue (low expression) (**f**–**i**) In situ hybridization (*Axin2*), qPCR *Axin2* and *Lef1*, and immunohistochemistry (AXIN2, LEF1, and SOX9) reveal upregulation of AXIN2 and LEF1 and downregulation of SOX9 in the Lmx1aCre::β-cat GOF ChPe compared with controls. (**f**, **h**) Representative images of sections taken from N = 3 brains (biologically independent replicates) examined over 2 independent experiments. Bar graphs (**g**) represent mean ± SEM, *N* = 3 brains (biologically independent replicates) for each genotype examined over 2 independent experiments. (**i**) Violin plots quantifying the nuclear intensity of LEF1 and SOX9, for LEF1: *n* = 505 nuclei for control and 500 nuclei for Lmx1aCre::β-cat GOF ChPe, *N* = 5 brains (biologically independent replicates) for each genotype examined over 3 independent experiments; color key: dark blue (grey value =0) and white (grey value =255). For SOX9, *n* = 457 nuclei for control and 444 nuclei for Lmx1aCre::β-cat GOF ChPe; *N* = 4 brains (biologically independent replicates) examined over 2 independent experiments. For violin plots (**c, i**) solid black line represents median and dotted lines represent quartiles). Statistical tests: Two-tailed unpaired Student’s *t* test with unequal variance (**c, g**), *p* < 0.0001 (**c**), *p* = 0.0005 (**g**, *Lef1)* and *p* = 0.0003 (**g**, *Axin2)*; two-tailed unpaired multiple Student’s *t* test with unequal variance (**i**), *p* < 0.000001 (**i**, LEF1 and SOX9). *p < 0.05, **p < 0.01, ***p < 0.001, ns if *p* value > 0.05. Scale bars: 10 μm (all panels in **a**, **b**, **f** and **h**); 100 μm (all panels in **d**). Source data are provided as a Source Data file.

*Lmx1a* expression is specific to the hem and the ChPe within the telencephalon^24^. To genetically manipulate the developing ChPe we used the Lmx1aCre line, which faithfully labels the hem lineage that is composed of Cajal-Retzius cells and ChPe cells, when crossed to the Ai9 reporter line^25^ (Fig. 2a). Therefore, the Lmx1aCre line offers the opportunity of disrupting genetic mechanisms not only in the ChPe, but also in its domain of origin, the hem.

We used Lmx1aCre together with two well-described mouse lines to introduce either gain-of-function (GOF) or loss-of-function (LOF) genetic perturbations in the *β-catenin* (*Ctnnb1*) gene. In *β-catenin* GOF mice, exon 3 of *β-catenin* is flanked by loxP sites, and Cre-mediated recombination results in deletion of a domain that contains phosphorylation sites for GSK3*β*, which would normally tag β-CATENIN for degradation. The recombined *β-catenin* allele encodes a protein that remains constitutively active^26^ (gain of function). This results in increased accumulation of active β-CATENIN within the nucleus of ChPe cells consistent with its role as a transcription factor^27^. Intensity quantification of the immunofluorescence within the nuclei of ChPe cells revealed that GOF mice have higher levels of active nuclear β-CATENIN than controls (Fig. 2b, c).

We examined whether these perturbations resulted in transcriptional changes that are typical of canonical Wnt pathway activity. We microdissected the ChP from E14.5 control and β-catenin GOF embryos, using the expression of the Ai9 reporter (Fig. 2d). This gave us a relatively pure ChP preparation that was processed for bulk RNA-seq. 2439 genes were downregulated and 2163 genes upregulated in the ChP upon constitutive activation of β-CATENIN, using a 1.5-fold cutoff (*p*.adj < 0.05; Supplementary Table 1). KEGG pathway analysis identified the Wnt pathway as one of the major pathways dysregulated in the Lmx1aCre::β-catenin GOF ChP (Supplementary Fig. S1). Consistent with this, multiple canonical Wnt pathway targets were positively regulated (*Ccnd1, Cdx1, Cldn1, c-Myc, Dkk2, Jun, Lef1, Met, Mycbp2, Neurod1, Pitx2, Sfrp2, Sox17, Tcf1, Wif1, Wnt4*), and others were negatively regulated (*Sostdc1, Sox9*,) (Fig. 2e; Supplementary Fig. S1;^28,29^, https://web.stanford.edu/group/nusselab/cgi-bin/wnt/target_genes). We further examined well-established positively regulated targets of this pathway, *Axin2*, *Lef1*, and a negatively regulated target *Sox9*, by in situ hybridization, qPCR, and/or immunohistochemistry, in the control and Lmx1aCre::β-catenin GOF ChPe. Both mRNA and protein for *Axin2/* AXIN2 and *Lef1/* LEF1 showed a marked increase in the GOF ChPe compared with controls. In contrast, SOX9, which is normally present in the ChPe, is greatly reduced in the β-catenin GOF ChPe, consistent with its suppression by canonical Wnt signaling^30^ (Fig. 2f–i).

We also examined the loss of function of β-CATENIN using Lmx1aCre using a well-described mouse line in which exon 2- 6 of the *ß-catenin* gene encoding a portion of the armadillo repeat region is removed by Cre-mediated recombination, resulting in a non-functional, transcriptionally inactive protein^23,31^. In the Lmx1aCre::β-catenin LOF ChPe cell nuclei, the levels of non-phosphorylated (active) β-CATENIN are nearly undetectable (Supplementary Fig. S2a, b). Nuclear LEF1 protein levels decreased compared with controls (Supplementary Fig. S2c, d). Furthermore, while TTR is present throughout the LOF ChPe at E16.5 and at birth (Supplementary Fig. S2e, f), AQP1 is undetectable in several cells, suggesting a functional disruption of the ChPe (Supplementary Fig. S2g, h).

β-CATENIN plays an important role in cell adhesion in addition to its function in transcriptional regulation. Consistent with this, both the GOF and LOF perturbations of β-CATENIN caused the ChPe to exhibit an abnormal morphology. The β-catenin GOF ChP was extensively folded, accompanied by a highly disorganized E-CADHERIN distribution (Supplementary Fig. S3a, b). In the β-catenin LOF ChP, even though the exons 2–6 that are deleted in the LOF allele are critical for its role in cell adhesion, the labeling pattern of E-CADHERIN appeared unaltered at E12.5. However, by birth, E-CADHERIN distribution was disorganized together with dysmorphia of the ChP, indicating that the loss of β-CATENIN may have a progressive effect on ChP morphogenesis with time in development (Supplementary Fig. S3c, d).

In summary, both GOF and LOF perturbations of β-CATENIN cause disruptions of the canonical Wnt pathway, with additional consequences on ChP morphology that are consistent with its role in cell adhesion. The level of nuclear β-CATENIN increased in the GOF and decreased in the LOF ChPe, and canonical Wnt targets displayed the expected effects as a result of these perturbations. We focused our subsequent analysis on the Lmx1aCre::β-catenin GOF ChPe in the mouse to examine the consequences of persistent activation of canonical Wnt signaling since this perturbation is similar to the observed pathological mutations in human β-CATENIN^20,21^. In a similar fashion, we also studied the effects of activated canonical Wnt signaling in hESC-derived organoids.

### Constitutively activated β-CATENIN causes a loss of ChPe identity

**Fig. 3:**
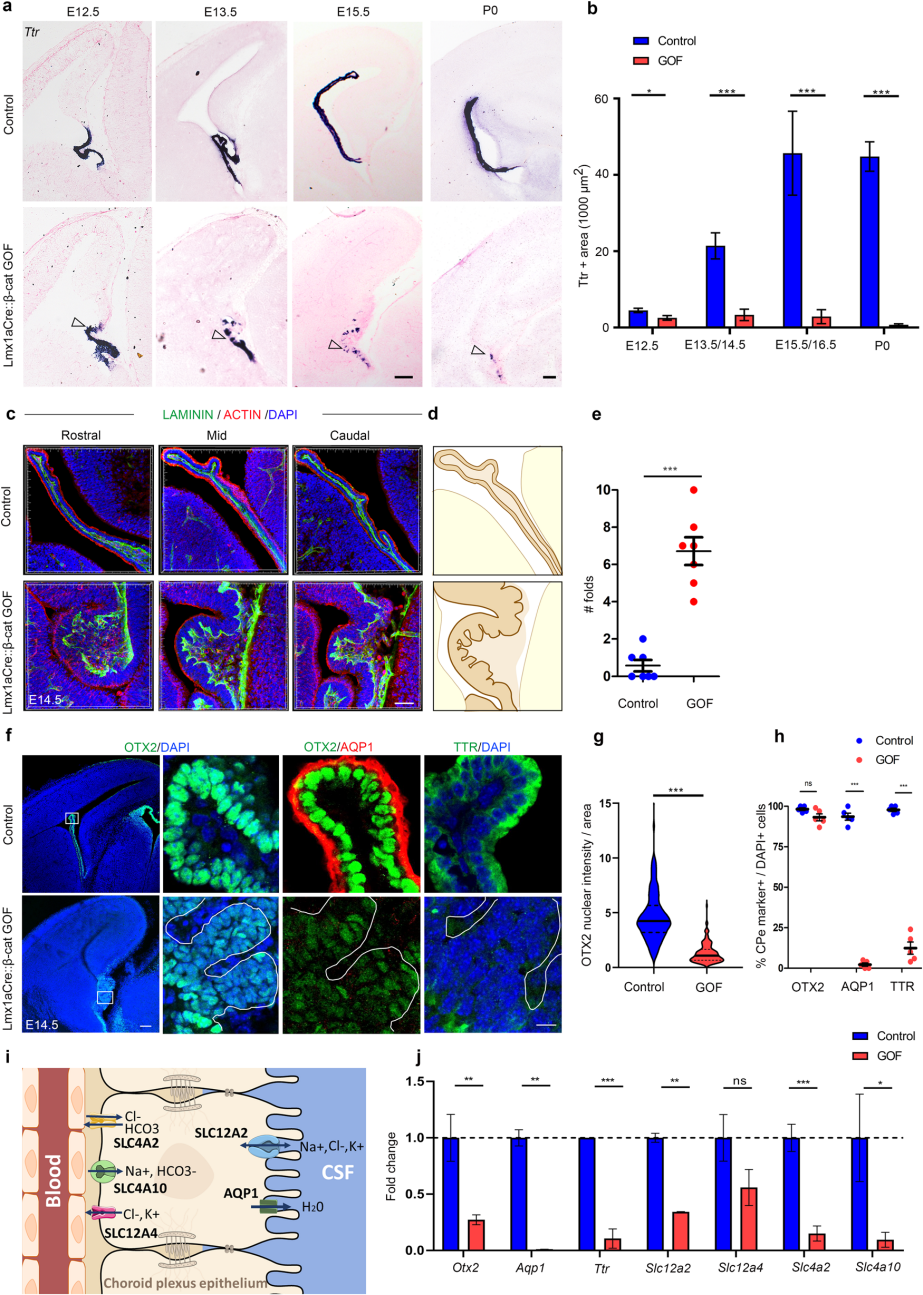
Constitutively active β-CATENIN in the hem and ChPe disrupts choroid plexus identity. **a**, **b** In situ hybridization for *Ttr* reveals a patchy expression (arrowheads) that is progressively lost from E12.5-P0 in Lmx1aCre::β-cat GOF brains compared with controls. **b** Quantification of *Ttr*-expressing area ± SEM, *N* = 4 (E12.5); *N* = 5 (E13.5/E14.5), *N* = 4 (E15.5/E16.5), *N* = 3 (P0) brains (biologically independent replicates) examined over 3 independent experiments. **c, d** Immunohistochemistry for ACTIN and LAMININ reveals that the ChP is extensively folded in Lmx1aCre::β-cat GOF brains compared with controls. **e** Quantification of the number of folds, *N* = 7 brains (biologically independent replicates) examined over 5 independent experiments. **f** ChPe markers, such as AQP1 and TTR are almost undetectable, and OTX2 labeling is greatly reduced in intensity in the Lmx1aCre::β-catenin GOF compared with controls. Boxed regions (**f**) are shown at high magnification in the adjacent panels. **g** Violin plot of OTX2 intensity/nuclear area reveals a significant reduction in the Lmx1aCre::β-cat GOF choroid plexus, *n* = 150 nuclei, *N* = 3 brains (biologically independent replicates) examined over 2 independent experiments. **h** Scatter plot corresponding to 500 DAPI + cells scored for the co-labeling for each choroid specific marker. In the control brains 98.2% cells are OTX2 + , 93.6% cells are AQP1 + , and 97.8% cells are TTR + whereas In Lmx1aCre::β-cat GOF ChPe 93.2% cells are OTX2 + , 2.3% cells are AQP1 + , and 12.4% cells are TTR + , *N* = 3 (**g**), *N* = 5 (**h**) brains (biologically independent replicates) examined over 3 separate experiments). **i** A cartoon illustrating transporters that are downregulated in the ChPe of β-cat GOF brains. **j** qPCR analysis of E14.5 ChP shows that not only *Ttr* and *Otx2* but also several transporters and channels such as *Aqp1, Scl12a2 (Nkcc1), Slc4a10 (NBCn2), Slc4a2 (Ae2)* are significantly downregulated in the Lmx1aCre::β-cat GOF ChP compared with control, *N* = 3 brains (biologically independent replicates) examined over 2 independent experiments. Solid black line represents median and dotted lines represents quartiles (**g**), bars (**b, e, h** and **j**) represent mean ± SEM. Statistical tests in **b** and **h**: Two-tailed unpaired multiple Student’s *t* test with unequal variance; for bar graph in *b*, *p* = 0.023685 (E12.5), *p* = 0.00002 (E13.5/14.5), *p* = 0.000777 (E15.5/16.5), *p* < 0.000001 (P0); statistical test in **e**, **g** and **j**: Two-tailed unpaired Student’s *t* test with unequal variance, *p* = 0.0001 (**e**), *p* < 0.0001 (**g**), *p* = 0.061246 (**h**, OTX2), *p* < 0.00001 (**h**, AQP1 and TTR), for (**j**) *p* = 0.0029 (*Otx2*), *p* = 0.0081 (*Aqp1*), *p* = 0.0005 (*Ttr*), *p* = 0.0048 (*Slc12a2*), *p* = 0.072 (*Slc12a4*), *p* = 0.0001 (*Slc4a2*), *p* = 0.0268 (*Slc4a10*). *p < 0.05, **p < 0.01, ***p < 0.001, ns if *p* value > 0.05. Scale bars: 100 μm (all panels in a and **f** low mag); 50 μm (**c**); 10 μm (all panels in f high mag). Source data are provided as a Source Data file.

To study the effect of constitutive activation of β-CATENIN on ChP development, we first examined an age series and found a progressive loss of *Ttr* expression in the Lmx1aCre::β-catenin GOF ChPe from E12.5 to birth (Fig. 3a, b). Immunostaining for apically enriched ACTIN and basal lamina marker LAMININ revealed major morphological changes from E14.5 in the form of multiple folds (Fig. 3c–e). Although OTX2, a transcription factor critical for ChPe development, was detected in 93.2% of the GOF ChPe cells (compared with 98.2% in the control), quantifications indicated that its level was drastically reduced in the GOF ChPe (Fig. 3f, g). AQP1 and TTR staining were undetectable in the vast majority of GOF ChPe cells, with only 2.3% or 12.4% cells positive for AQP1 or TTR, respectively, compared with 93.6 and 97.8%, respectively, in the controls (Fig. 3f, h). The ventricles of Lmx1aCre::β-catenin GOF brains collapsed as development proceeded (Fig. 3a), possibly due to a lack of fluid resulting from reduced AQP1 together with a reduction in several solute transporters encoded by the SLC family (*SLC12a2, 4a2, 4a10*; Fig. 3i, j). The progressive nature of the phenotype is consistent with the constitutive and persistent nature of the GOF disruption of the *β-Catenin* gene.

### Gain of neuronal identity in the ChPe upon constitutive activation of β-CATENIN

**Fig. 4:**
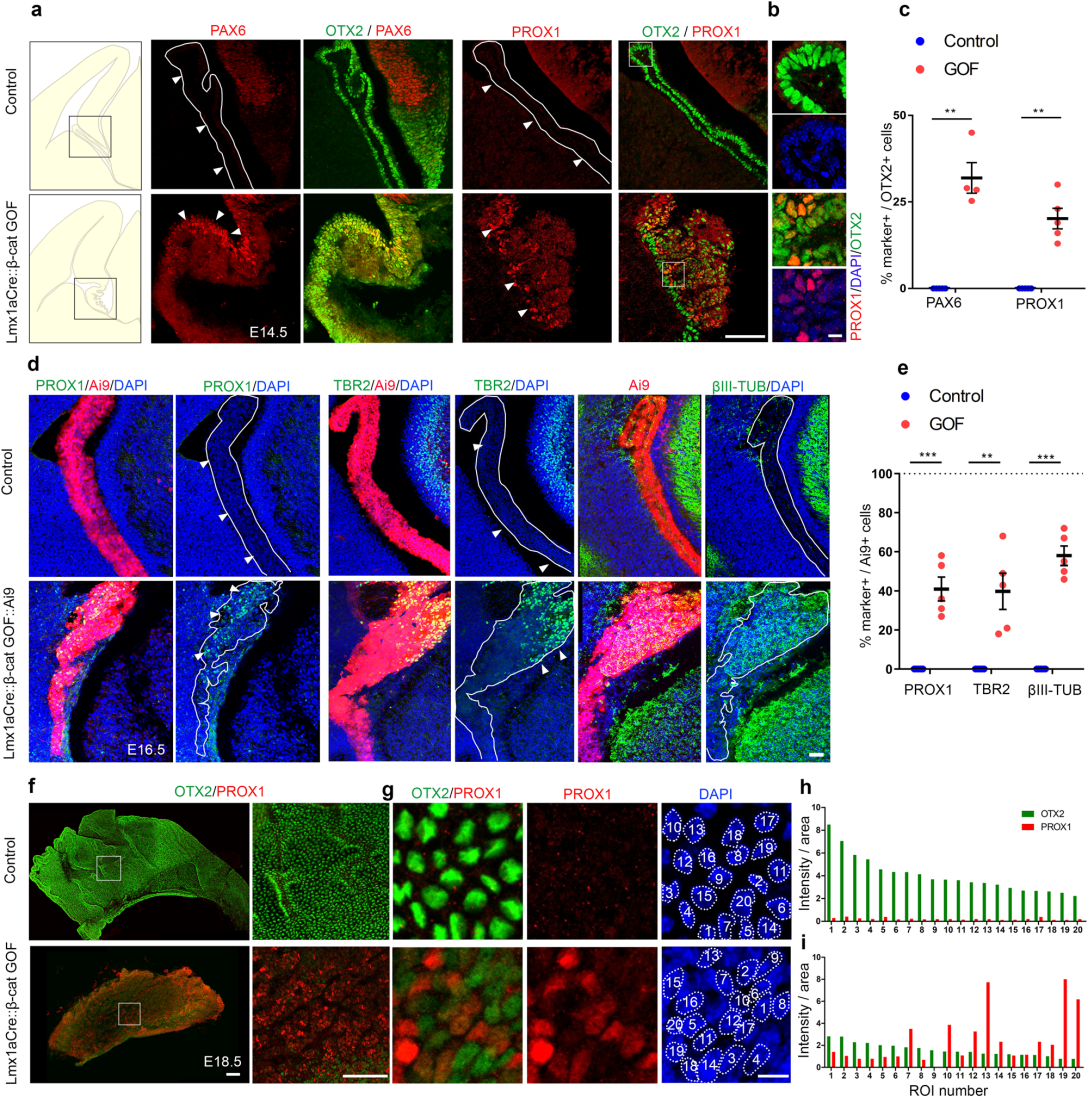
Constitutive activation of β-catenin transforms the ChPe to a neuronal and hippocampal-like molecular identity. (**a**–**c**) At E14.5, the control ChPe is positive for OTX2 but not PAX6 or PROX1. In the control brains 0% OTX2 + cells are also PAX6 + / PROX1 + whereas in Lmx1aCre::β-cat GOF ChPe, 31.9% of the OTX2 + cells are also PAX6 + and 20.3% are PROX1 + . Boxed regions (**a**) are shown at high magnification in the adjacent panels (**b**). (**c**) *N* = 4 (PAX6), *N* = 5 (PROX1) brains (biologically independent replicates) for each genotype were examined over 4 independent experiments. (**d**, **e**) At E16.5, the Ai9 reporter marks the Lmx1aCre lineage, none (0%) of Ai9+ control ChPe cells are PROX1 + or TBR2 + or, βIII TUBULIN + . In Lmx1aCre::β-cat GOF brains, 41% of the Ai9+ cells co-label for PROX1, 39.8% for TBR2, and 58% for βIII TUBULIN. (**e**) *N* = 5 brains (biologically independent replicates) for each genotype were examined over 5 independent experiments. (**f**, **g**) E18.5 control and Lmx1aCre::β-cat GOF wholemount ChP preparations were co-immunostained for PROX1 and OTX2. The Lmx1aCre::β-cat GOF ChP displays decreased OTX2 labeling and the presence of PROX1 + cells of varying intensities which never appear in control samples. (**h, i**) Intensity quantification of 20 cells from a single field shows a marked decrease in OTX2 intensity in cells from the Lmx1aCre::β-cat GOF ChPe, and high levels of PROX1 in some of these cells. (**f**, **g**) Representative images of sections taken from *N* = 3 brains (biologically independent replicates) examined over 2 independent experiments. High mag panels in (**f**) show representative z plane of the boxed region. Statistical tests in **c** and **e**: two-tailed unpaired multiple Student’s *t* test with unequal variance. For (**c**), *p* = 0.005442 (PAX6*)*, *p* = 0.0023 (PROX1); (**e**), *p* = 0.000156 (PROX1*)*, *p* = 0.00264 (TBR2), *p* = 0.000003 (βIII TUB). *p < 0.05, **p < 0.01, ***p < 0.001, ns if *p* value > 0.05. Scatter plots (**c**, **e**) represent mean ± SEM. Scale bars: 100 μm (all panels in **a** and **f**); 10 μm (all panels in **b** and **g**); 50 μm (all panels in d). Further information on replicates and reproducibility for this figure is mentioned in the “Statistics and Reproducibility” section of the Methods. Source data are provided as a Source Data file.

The loss of ChPe markers in Lmx1aCre::β-catenin GOF brains prompted us to examine whether this tissue acquired a different identity. It is well established that canonical Wnt signaling from the cortical hem is necessary and sufficient for the induction of hippocampal fate in the adjacent neuroepithelium^32–34^. Therefore, we tested whether neuronal, and in particular hippocampal markers were upregulated in the Lmx1aCre::β-catenin GOF ChPe. Remarkably, at E13.5, parts of the GOF ChPe were positive for both the ChPe marker OTX2, and PAX6 which is normally restricted to neuronal ventricular zone progenitors (Fig. 4a; Supplementary Fig. S4). Likewise, several cells were positive for OTX2 as well as the dentate granule cell marker PROX1. Overall, 31.9% OTX2 + cells were PAX6 + and 20.3% were PROX1 + in the β-catenin GOF ChPe, compared with no co-labeling (0%) for each of these markers in controls (Fig. 4a–c). The β-catenin GOF also displays enhanced proliferation as seen by PH3 labeling, and apoptosis as detected by staining for cleaved CASPASE 3 (Supplementary Fig. S4).

By E16.5, 41% of Cre-expressing (Ai9) cells were positive for PROX1 (Fig. 4d, e) in Lmx1aCre::β-catenin GOF brains compared with 0% in the controls. As development proceeds, the telencephalic ventricular zone progenitors give rise to a TBR2-positive pool of sub-ventricular zone progenitors. At E16.5, the GOF ChPe displayed this feature as well, such that numerous (39.8%) TBR2-positive cells were scattered in this tissue, but were never seen in controls (Fig. 4d, e). In addition, the postmitotic neuronal marker βIII TUBULIN appeared in 58% of the Ai9+ cells in the GOF ChPe compared with 0% in controls (Fig. 4d, e). The ChP is normally populated by a small number of scattered neuronal cells^35^, which also appeared in the control sections we examined. However, these do not arise from the hem and did not express Ai9, whereas the TBR2 and βIII TUBULIN positive cells in the GOF ChPe co-localized with Ai9 (Supplementary Fig. S5). PROX1 labeling persisted in the Lmx1aCre::β-catenin GOF at E18.5. While PROX1 was never detected in the controls, it was present in varying intensities in the cells of the GOF ChPe in which OTX2 levels were uniformly low compared with controls (Fig. 4f–i).

**Fig. 5:**
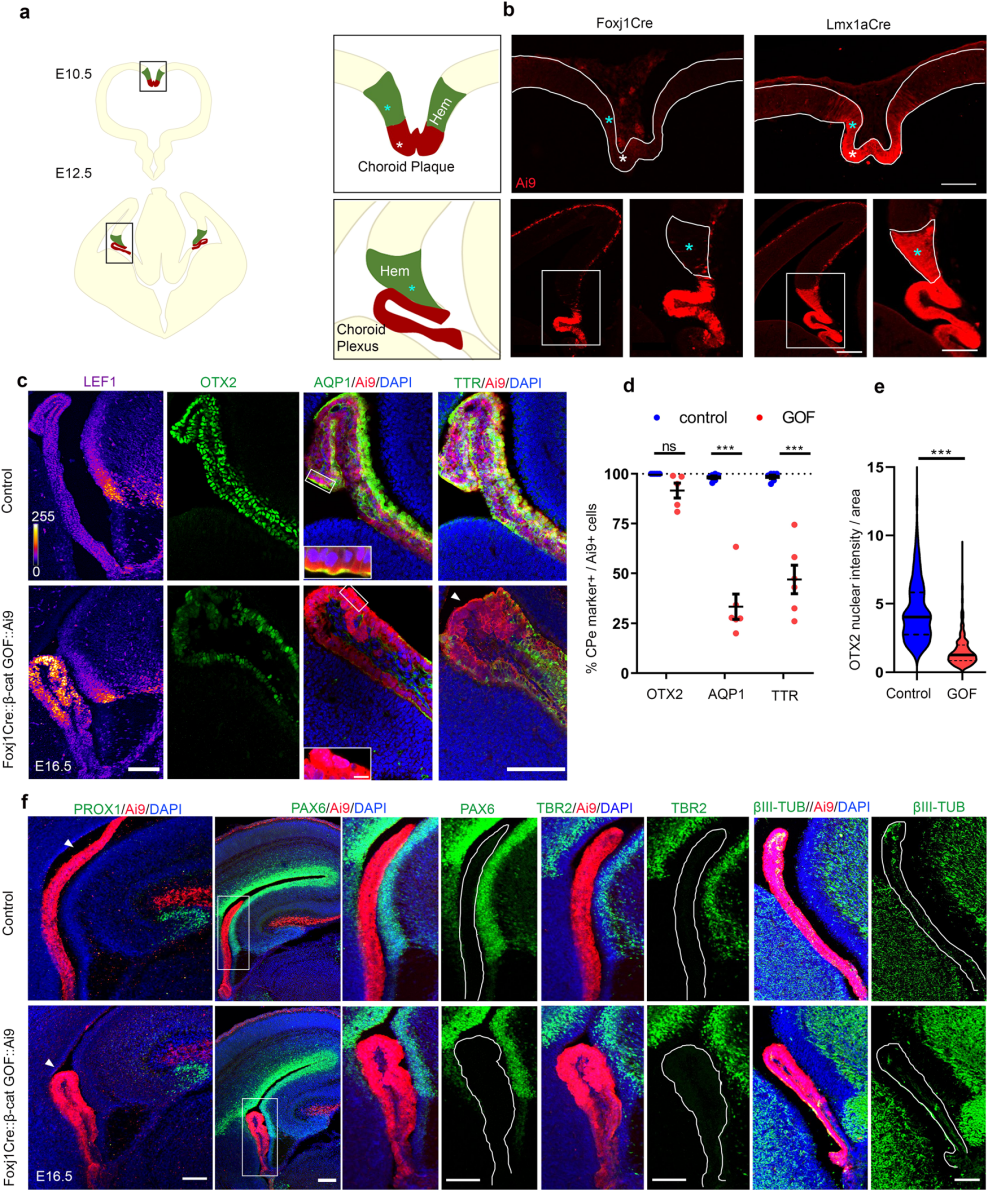
Stabilizing β-CATENIN selectively in the specified ChPe using FoxJ1Cre causes loss of ChPe fate but not transformation to a neuronal fate. **a** A cartoon illustrating the hem (cyan asterisks), choroid plaque (white asterisks), and choroid plexus at E10.5 and E12.5. **b** Foxj1Cre::Ai9 marks differentiated choroid plexus at E12.5 but not the choroid plaque at E10.5 or the hem at either stage. Lmx1aCre::Ai9 marks both hem and choroid plexus/plaque starting from E10.5. Boxed regions (**b**) are shown at high magnification in the adjacent panels. Representative images are shown of sections taken from N = 3 brains (biologically independent replicates) examined over 2 independent experiments. **c**–**e** In E16.5 Foxj1Cre::β-cat GOF embryos, LEF1 levels increase (color key: dark blue indicates grey value =0, white indicates grey value =255), while bonafide ChPe markers AQP1(Boxed regions (**c**) are shown as high magnification insets), TTR, and OTX2 decrease compared with controls, as seen using (**c**) immunofluorescence and (**d**) quantification of the number cells positive for each ChPe marker as a fraction of the Ai9-positive cells. A scatter plot shows that in the E16.5 control ChPe, 100% of 500 Ai9+ cells are also OTX2 + , 98.25% of 942 Ai9+ cells are also AQP1 + , and 98.47% of 1168 Ai9+ cells are also TTR + . In Foxj1Cre::β-cat GOF ChP, 91.6% of 731 Ai9+ cells are also OTX2 + , 33.3% of 781 Ai9+ cells are also AQP1 + , and 47% of 1097 Ai9+ cells are also TTR + . (**c**, **d**) *N* = 5 (OTX2) and *N* = 6 (AQP1 and TTR) brains (biologically independent replicates) for each genotype examined over 3 independent experiments. Bars represent mean ± SEM; *p* = 0.088919 (OTX2), *p* = 0.000129 (AQP1), *p* = 0.000783 (TTR). (**e**) A violin plot reveals that the nuclear intensity of OTX2 labeling decreases significantly in Foxj1Cre::β-cat GOF ChPe cells compared with controls. n = 536 nuclei from control and 590 nuclei from Foxj1Cre::β-cat GOF ChPe; *N* = 5 brains (biologically independent replicates) examined over 3 independent experiments; solid black line represents median and dotted lines represents quartiles; statistical test: two-tailed unpaired multiple Student’s t test with unequal variance; *p* < 0.0001; *p < 0.05; **p < 0.01; ***p < 0.001; ns if *p* value > 0.05. **f** In control and Foxj1Cre::β-cat GOF brains, PROX1 immunostaining is seen in the dentate gyrus, PAX6 and TBR2 appear in the telencephalic ventricular and sub-ventricular zones, respectively, and βIII-TUBULIN is present in postmitotic neurons in the dorsal telencephalon. None of these markers appear in the ChPe of either genotype (solid lines), except for a few βIII-TUBULIN positive neurons that normally populate the ChPe. Boxed regions (**f**) are shown at high magnification in the adjacent panels. Representative images are shown of sections taken from *N* = 3 brains (biologically independent replicates) examined over 2 independent experiments. Scale bars: 100 μm (all panels in **b**, **c**, and **f**); 10 μm (all panel in **c** inset). Source data are provided as a Source Data file.

Lmx1aCre acts in the progenitor domain for the ChPe, the cortical hem, as well as in the differentiated ChPe^25^. We compared this line with Foxj1Cre, which acts in ciliated cells in the brain, and is known to be expressed in the primordial ChPe from E11.5^36,37^. When each Cre line was crossed to the Ai9 reporter, the differences in their expression domain were apparent: Foxj1Cre did not appear to be active at E10.5, at which stage Lmx1aCre was expressed in the hem and in the choroid plaque (arrowheads, Fig. 5a, b), which is the precursor of differentiated ChPe. At E12.5, Foxj1Cre was expressed in the ChPe and by Cajal-Retzius cells, but excluded from the hem, whereas Lmx1aCre was expressed in all these structures (Fig. 5a, b). Foxj1Cre::β-catenin GOF embryos displayed a dysmorphic ChP that recapitulated the loss of ChPe identity seen in Lmx1aCre::β-catenin GOF brains (Fig. 5). At E16.5, only 33.3% of the Ai9 cells were also AQP1-positive and 47% were TTR positive, compared with 98.25 and 98.47%, respectively, in the controls. While OTX2 labeling persisted in the Foxj1Cre::β-catenin GOF ChPe, its level was significantly lower than in controls (Fig. 5c–e), mirroring that seen in Lmx1aCre::β-catenin GOF brains. These changes were apparent throughout the rostro-caudal extent of the Foxj1Cre::β-catenin GOF ChPe (Supplementary Fig. S6). Therefore, the loss of ChPe identity in the Foxj1Cre::β-catenin GOF ChPe recapitulated that seen in the Lmx1aCre::β-catenin GOF ChPe.

In contrast, the Foxj1Cre::β-catenin GOF ChPe failed to display any transformation to a neuronal fate. None of the markers we reported in the Lmx1aCre::β-catenin GOF ChPe, i.e., PROX1, PAX6, TBR2, or βIII TUBULIN, were detected in the Foxj1Cre::β-catenin GOF ChPe (Fig. 5f). This offers an insight into the timing and stage at which the canonical Wnt pathway can modulate neuronal fate specification in cell types that normally take on ChPe fate.

**Fig. 6:**
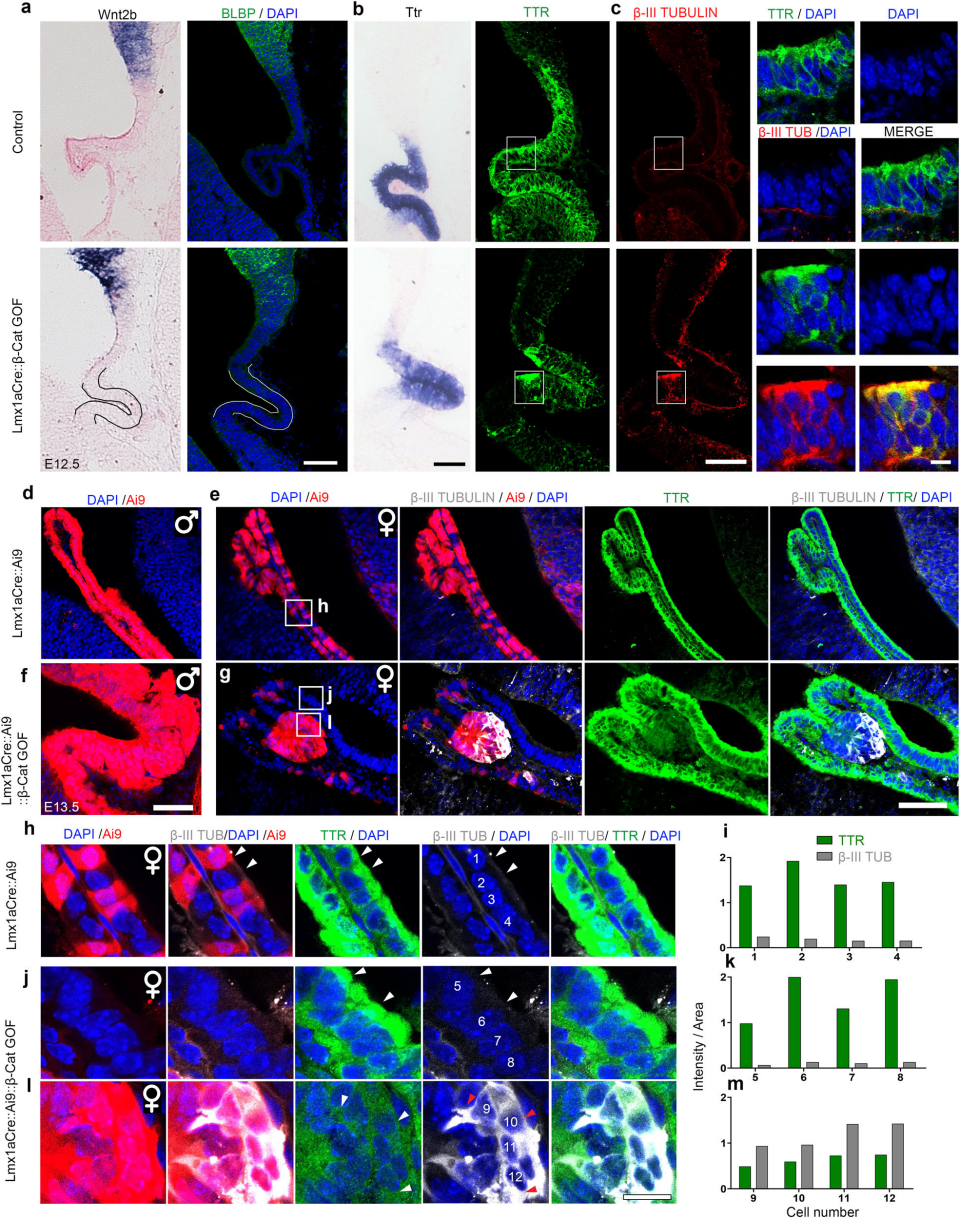
β-Catenin GOF causes upregulation of β-III TUBULIN in TTR + ChPe cells. (**a**–**c**) At E12.5, both control and Lmx1aCre::β-Catenin GOF ChP display a ribbon-like morphology and do not express hem markers *Wnt2b* and BLBP (**a**) but express *Ttr* mRNA and TTR protein (**b**). (**c**) Co-immunostaining for β-III TUBULIN and TTR identifies cells that display both markers in the β-Catenin GOF but not in the control ChPe. (**a**–**c**) Representative images are shown of sections taken from *N* = 3 brains (biologically independent replicates) examined over 2 independent experiments. Boxed regions (**c**) are shown at high magnification in the adjacent panels. (**d**–**g**) At E13.5, male Lmx1aCre::Ai9 control and Lmx1aCre::Ai9::β-Catenin GOF brains display Cre-driven Ai9-positive cells throughout the ChPe (**d**, **f**), In contrast, female control and β-Catenin GOF brains show mosaic Ai9 due to random X-inactivation (**e**, **g**). β-III TUBULIN is undetectable in the female control ChPe, but appears selectively in the Ai9+ patches in the female β-Catenin GOF ChPe (**e**, **g**). (**h**–**k**). In females, neither control Lmx1aCre::Ai9 ChPe cells (cells #1–4; **h**, **i**) nor non-Ai9 cells internal control cells in Lmx1aCre::Ai9::β-Catenin GOF ChPe (cells #5–8; **j**, **k**) display co-localization of β-III TUBULIN and TTR. These markers are both detected in Ai9-positive Lmx1aCre::Ai9::β-Catenin GOF ChPe cells (#9–12; l, **m**). (**d**–**l**), Representative images are shown of sections taken from *N* = 3 brains (biologically independent replicates) examined over 2 independent experiments. (**i**, **k**, **m**) Fluorescence intensity quantifications of cells #1–12 from (**h**, **j**, **l**). Boxed regions (**e**, **g**) are shown at high magnification in the panels (**h**, **j**, **l**) below. Scale bars: 10 μm (all high mag panels in **c** and all panels in **h**–**l**); 50 μm (all panels in **a**–**e**). Further information on replicates and reproducibility for this figure is mentioned in the “Statistics and Reproducibility” section of the Methods. Source data are provided as a Source Data file.

Since the loss of ChPe identity was accompanied by a gain of neuronal identity only when the β-catenin GOF was initiated in the hem, it was important to examine whether ChPe progenitors were converted to neuronal progenitors or whether specified ChPe cells that displayed ChPe markers were transformed to a neuronal fate. Therefore, we examined whether neuronal markers appear in cells that also display ChPe markers. At E12.5, both control and Lmx1aCre::β-catenin GOF brains displayed *Wnt2b* and BLBP restricted to the hem while *Ttr* mRNA and TTR protein was restricted to the ChPe (Fig. 6a, b). Whereas βIII TUBULIN staining was almost undetectable in control ChPe, a small region of the β-catenin GOF ChPe was βIII TUBULIN positive, co-localizing with TTR immunostaining (Fig. 6c). This indicated that neuronal markers were upregulated in specified ChPe cells from early stages in its development.

To examine this neuronal transformation further, we took advantage of X-inactivation-based mosaic expression of the Cre recombinase in Lmx1aCre female embryos. Whereas male brains expressed the Ai9 reporter in the entire *Lmx1a* expression domain (Fig. 6d, f), female Lmx1aCre brains displayed a mosaic expression of the Ai9 reporter (Fig. 6e, g), in both control (Lmx1aCre::Ai9) and Lmx1aCre::Ai9::β-catenin GOF animals. In control female brains, all ChPe cells, whether Ai9 positive and negative, were TTR positive but did not display detectable βIII TUBULIN immunofluorescence (Fig. 6e, h). In β-catenin GOF female brains, ChPe cells that were Ai9-negative (no Cre activity) were indistinguishable from controls (Fig. 6g, j). Consistent with the findings in male β-catenin GOF brains, some Ai9-positive (Cre-active) cells in the ChPe of female β-catenin GOF brains displayed βIII TUBULIN immunofluorescence in cells that were also TTR positive (Fig. 6g, l). Similar results were seen when ChPe marker E-CADHERIN was examined together with βIII TUBULIN in female β-catenin GOF brains (Supplementary Fig. S7a): ChPe cells that were Ai9-negative (no Cre activity) never displayed βIII TUBULIN staining (Supplementary Fig. S7b); whereas some Ai9-positive (Cre-active) cells were positive for both E-CADHERIN and βIII TUBULIN labeling (Supplementary Fig. S7c). In summary, the mosaic expression of the Cre recombinase in female embryos offered an elegant internal control in which Cre-driven β-catenin GOF cells resided adjacent to control (no Cre activity) cells in the same ChPe tissue. These data demonstrated that ChPe cells that were TTR or E-CADHERIN positive also expressed βIII TUBULIN in β-catenin GOF brains, and therefore led to the conclusion that cells that were specified as ChPe became transformed to a neuronal fate. The observation that βIII TUBULIN positive cells were always Ai9 positive further suggests the effect of β-catenin GOF was likely to be cell-autonomous.

**Fig. 7:**
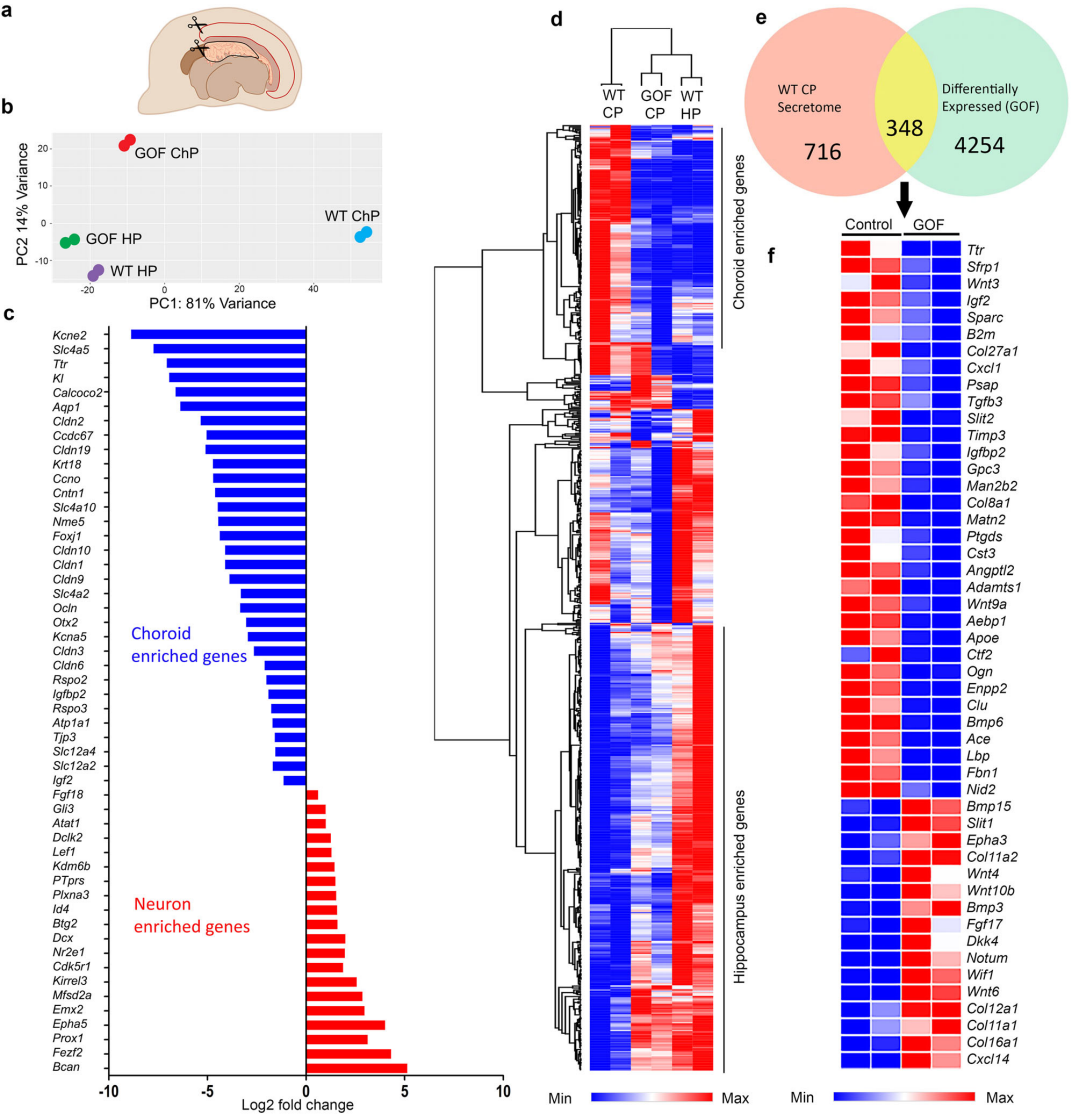
Constitutive activation of β-CATENIN leads to a dysregulation of the ChP transcriptome. (**a**) A cartoon depicting the microdissection E14.5 ChP and hippocampus for RNAseq analysis. (**b**) Principal Component Analysis (PCA) of the RNAseq datasets. (**c, d**) Choroid plexus-enriched genes are downregulated, and hippocampus/neuron-enriched genes are upregulated in the Lmx1aCre::β-catenin GOF ChP. In (**c**) log2 fold changes of 31 representative choroid plexus-enriched genes and 20 neuron-enriched genes are plotted (Padj < 0.05), (**d**) heatmap and hierarchical clustering show read counts of 500 choroid enriched and 500 hippocampus enriched genes from the RNA-seq dataset (Supplementary data 1). (**e, f**) Venn diagram showing the overlap between genes corresponding to the ChP secretome (from Lun et al. 2015.) and differentially expressed genes in the Lmx1aCre::β-catenin GOF versus control ChP and heat map of selected genes plotted as read counts (**e**) shows the dysregulated genes belong to various known pathways of cell-cell signaling (**f**). Color bars in (**d, f**): blue (low expression), red (high expression). For RNAseq experiments (**c, d,** and **f**) *N* = 2 biologically independent replicates. Further information on replicates and reproducibility for this figure is mentioned in the “Statistics and Reproducibility” section of the Methods and Supplementary data 1. Source data are provided as a Source Data file.

To examine the nature of this apparent neuronal transformation of the Lmx1aCre::β-catenin GOF ChP in a comprehensive manner, we compared transcriptomic datasets obtained from microdissected E14.5 hippocampal primordia isolated from the same telencephalic hemispheres from which the ChP was isolated for RNAseq (Fig. 2), as illustrated in the cartoon (Fig. 7a; Supplementary Data 1). Principal Component Analysis (PCA) of the overall transcriptomes revealed that the Lmx1aCre::β-catenin GOF ChP is more similar to the control and GOF hippocampus than it is to the control ChP (Fig. 7b). Consistent with this, the expression of genes critical for ChP development and function, such as *Aqp1, Htr2c, Kcne2, Otx2, Slc12a2(Nkcc1)*, are dramatically reduced, and the expression of genes important for neuronal development such as *Emx1, Emx2, Fezf2, Neurod1, Prox1* are upregulated in the β-catenin GOF ChP (Fig. 7c). Hierarchical clustering of the top 500 genes expressed in the ChP and the hippocampus recapitulates this loss of choroid epithelial fate and gain of neuronal fate in the Lmx1aCre::β-catenin GOF ChP (Fig. 7d; Supplementary Data 1).

**Fig. 8:**
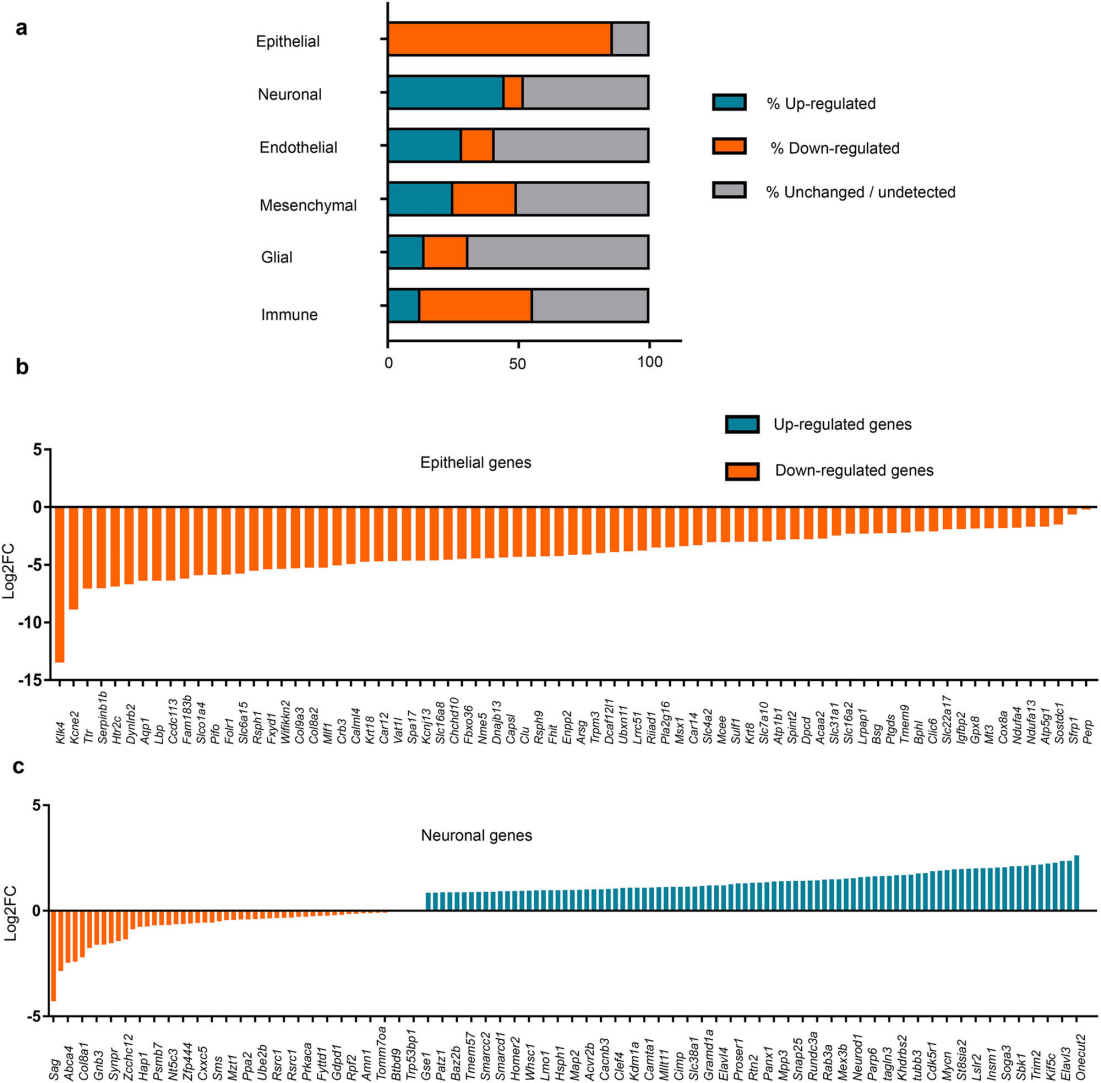
Cell-type classification of genes dysregulated upon constitutive activation of β-CATENIN. (**a**) comparison of RNA-seq data from E14.5 Lmx1aCre::β-catenin GOF ChP with scRNA-seq data from E16.5 ChP (Dani et al., 2021). Genes that were differentially expressed (DE) between the Lmx1aCre::β-catenin GOF ChP and control ChP (Supplementary data 1) were compared with the genes enriched in Epithelial/Neuronal/Endothelial/Mesenchymal/Glial/Immune cell groups identified in Dani et al., 2021. For each cell group, the DE list contained genes that were either upregulated (blue), downregulated (orange), or unchanged/undetected (grey). (**b, c**) Log2 fold change (*P*adj < 0.05) selected choroid plexus epithelial genes (74 downregulated) and neuronal-enriched genes (122 upregulated and 21 downregulated) that were dysregulated in the Lmx1aCre::β-catenin GOF ChP upon comparison with Dani et al., 2021. Bar graphs are color coded as Upregulated (blue), downregulated (orange). For RNAseq experiments (**a, b,** and **c**) *N* = 2 biologically independent replicates. Further information on replicates and reproducibility for this figure is mentioned in the “Statistics and Reproducibility” section of the Methods and Supplementary data 1. Source data are provided as a Source Data file.

A recent study used single-cell RNAseq for transcriptomic analysis of the developing mouse ChP and identified five distinct cell types in this tissue, namely epithelial, neuronal, endothelial, mesenchymal, and immune cells. Dani et al., 2021^35^ identified 86 ChP epithelial cell-enriched genes in the ChP, and of these, 74 (86 %) were significantly downregulated in the Lmx1aCre::β-catenin GOF ChP, consistent with our interpretation of a loss of ChPe fate; none were upregulated; 1 gene did not change in expression, and 11 were not detected. In contrast, of the 274 neuronal-enriched genes, 123 (45 %) were upregulated in the Lmx1aCre::β-catenin GOF ChP, consistent with our interpretation of a gain of neuronal fate; 21 were downregulated and 122 did not change in expression; 9 were not detected (Fig. 8a–c; Supplementary Data 1). The genes upregulated in the GOF ChP included several known regulators of neuron differentiation such as bHLH members *Neurod1, Neurod2, Neurod6, Neurog2*, *Tcf3*, *Sox21, Sox4, Sox1, Zeb1,* and *Zeb2* (Fig. 8c, Supplementary Fig. S8).

Although the remaining cell types identified in Dani et al., 2021^35^ are not known to arise from the hem, it is intriguing that a substantial fraction of genes enriched in these populations were also dysregulated in the Lmx1aCre::β-catenin GOF ChP, suggesting the possibility of noncell autonomous effects of β-catenin GOF in the hem-derived cells. This further suggests interactions between these cell types that have thus far not been examined.

Consistent with the apparent neuronal transformation of the GOF ChP, gene ontology analysis of the Lmx1aCre::β-catenin GOF transcriptome indicated that genes that were upregulated in the GOF ChP belonged to the categories like neurogenesis (GO: 0022008), neuron projection development (GO: 0031175), cell differentiation (GO: 0030154), dentate gyrus development (GO: 0021766, GO: 0021542). We also examined GO categories in which genes were downregulated in the GOF ChP, and found that these belonged to classes important for ChP function, such as cilium organization (GO:0044782) and ion/solute transport (Supplementary Fig. S8d). We examined the effects of this transformation on the CSF in Lmx1aCre::β-catenin GOF brains. It was not possible to extract CSF from Lmx1aCre::β-catenin GOF brains because the ventricles were collapsed from E13.5 (Fig. 3a). Therefore, we took the alternative approach of examining a curated list of 1064 ChP-secretome genes from the published literature^38^ and comparing it with the set of genes differentially expressed in the GOF versus control ChP. 348 of the ChP-secretome genes were dysregulated in the GOF ChP (Fig. 7e). The majority (211/348) of these genes were downregulated compared with the control ChP, including several major components of the CSF such as *Igf2, Igfbp2, Ttr* (Fig. 7f). Some genes were upregulated in the GOF ChP, such as *Bmp6, Fgf17, Wnt4 and 6*, suggesting a change in the composition of the secreted CSF in Lmx1aCre::β-catenin GOF brains. Together these results suggest that the ChP has partially transformed, acquired neuronal characteristics, and the gene expression data suggest an altered secretome that would impact CSF composition.

### ChPe-like structures arise in hESC organoids upon a combination of BMP and canonical Wnt pathway activation

**Fig. 9:**
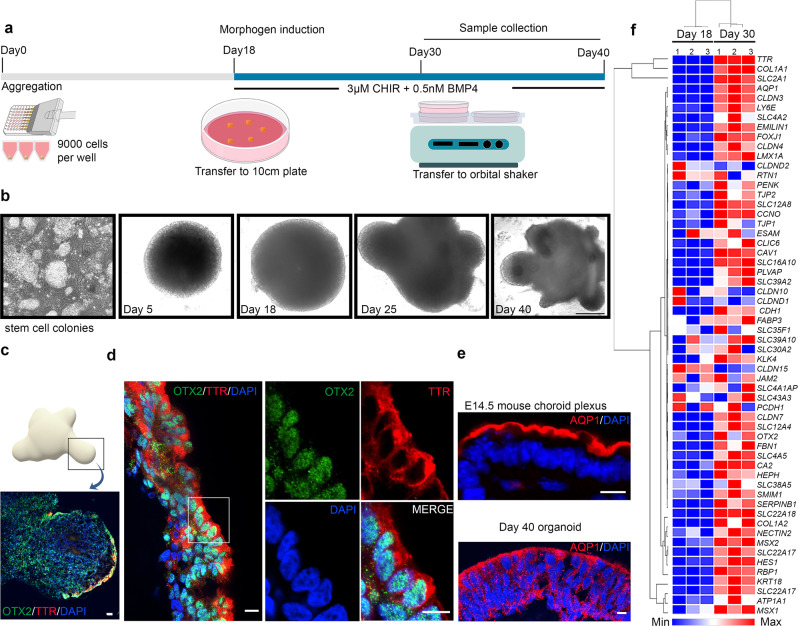
Differentiation of hESC organoid cultures using a ChPe differentiation-promoting protocol. **a** Schematic presentation describing the organoid culture protocol. (b) Bright-field images of organoids at different stages of development. Representative images from *N* = 48 organoids (biologically independent replicates) examined over 3 separate experiments. (**c, d**) Immunostaining reveals the presence of choroid markers OTX2 and TTR in the perimeter of the protrusions of a day 40 organoid. Boxed region (**c, d**) is shown at high magnification in the adjacent panels. (**e**) AQP1 immunostaining in Day 40 organoids is similar to that seen in the E14.5 mouse ChP. (**c–e**) Representative images of sections taken from *N* = 12 organoids (biologically independent replicates) examined in 2 separate experiments. (**f**) Heatmap and hierarchical clustering generated from normalized reads obtained by MARS-Seq show upregulation of ChP-enriched genes by day 30 of the organoid culture (*N* = 3 biologically independent replicates). Scale bars: 200 μm (all panels in **b**); 100 μm (**c**); 10 μm (all panels in d and **e**). Source data are provided as a Source Data file.

We sought to examine whether constitutive activation of canonical Wnt signaling disrupts ChPe identity in the human, as it does in the mouse. First, we used established protocols to achieve ChPe differentiation in hESC-derived organoids^39^. In this protocol, hESCs are aggregated and seeded in a 96 well low-adhesion plate, and over 18 days of incubation, they form spherical structures which grow in diameter (Fig. 9a, b). At this stage, a morphogen cocktail containing BMP4 and CHIR, a GSK3 inhibitor, is added. GSK3 is responsible for phosphorylating β-CATENIN and marking it for degradation, so CHIR addition results in a greater amount of active β-CATENIN^40^. However, unlike the β-catenin GOF mouse, CHIR can be added in graded amounts to control the extent of canonical Wnt pathway activation^41^. A cocktail containing a low level of CHIR (3 μM) together with BMP4 (0.5 nM) promotes ChPe-like differentiation^39^. The spherical organoids became slightly tapering and developed multiple protrusions within a few days of exposure to the cocktail (Fig. 9b). Immunohistochemistry revealed ChPe markers TTR, AQP1, and OTX2 were present along the perimeter of the protrusions, with cells displaying a cuboidal morphology (Fig. 9c–e). To better characterize these organoids, we performed RNA-seq after 12 days of exposure to the cocktail. Even though the ChPe-like differentiation was seen only along the perimeter of the organoid, the transcriptomic analysis of the sample revealed characteristic markers of the ChPe such as *AQP1, CLIC6, KRT18*, *and TTR* (Fig. 9f). Interestingly, genes associated with the blood-CSF barrier (BCSFB), such as *CDH1*, *CLDN3*, *JAM2, TJP1, and TJP2* and were also upregulated, as were several transporters and ion channels encoded by the SLC gene family and genes associated with ciliogenesis, consistent with the ciliated epithelium nature of the ChPe (Supplementary Fig. S9). In summary, consistent with Sakaguchi et al. (2015)^39^, ChPe-like structures develop in organoids upon exposure to a cocktail of BMP4 and low levels of CHIR.

### Loss of ChPe-like identity upon increased activation of canonical Wnt signaling in hESC-derived organoids

**Fig. 10:**
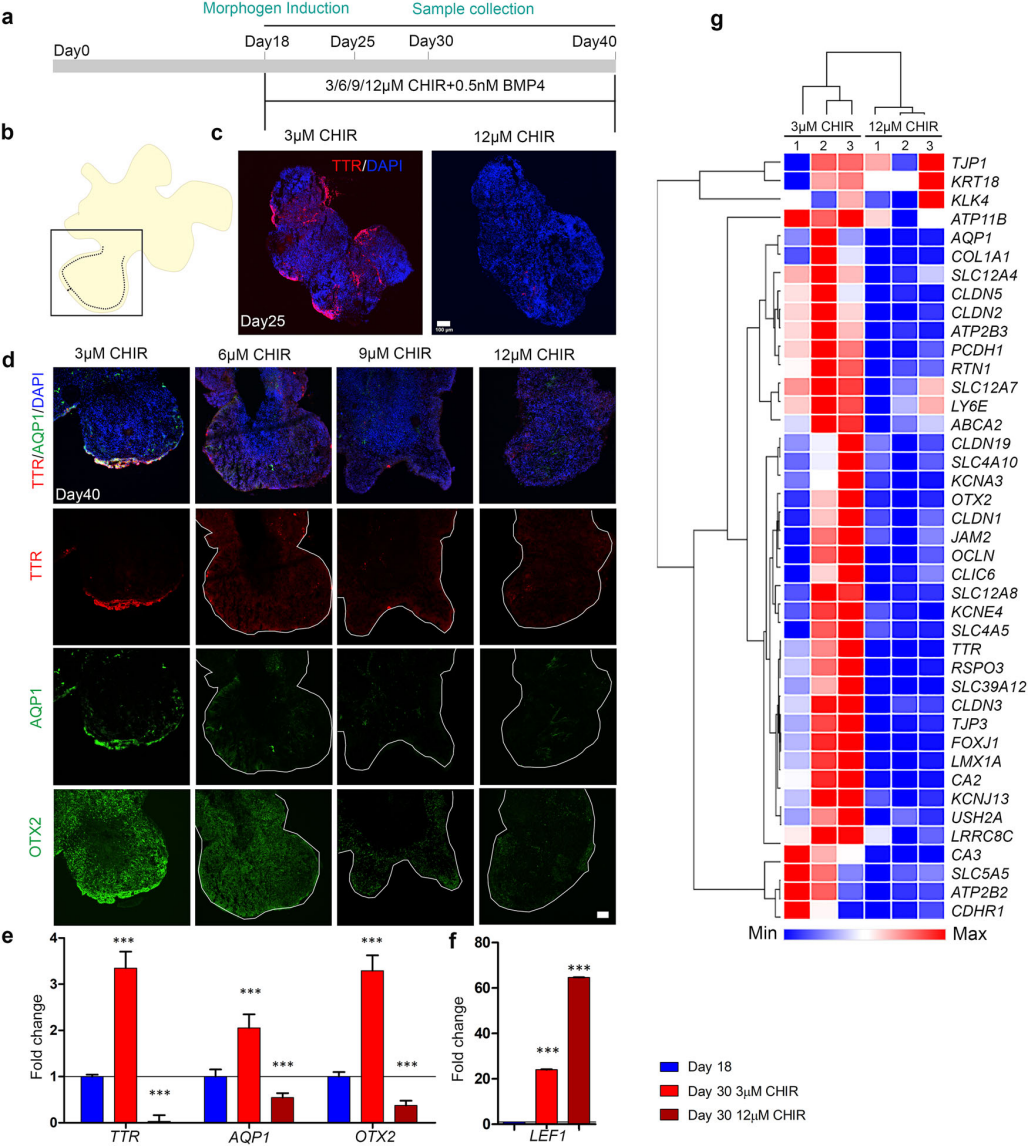
High activation of the canonical Wnt pathway in hESC-derived organoids causes suppression of ChPe markers. **a** A schematic presentation of the protocol for the treatment of organoids with the canonical Wnt agonist CHIR. **b**, **c** The ChP marker TTR is present in the periphery of a day 25 organoid exposed to 3 μM, but not when exposed to 12 μM CHIR, from day 18. **c** *N* = 4 organoids (3 μM CHIR) and 5 organoids (12 μM CHIR treated); were used for immunohistochemistry analysis. **d** High magnification images (boxed region shown in b) of serial sections of a day 40 organoid exposed to 3 μM reveal the presence of TTR, AQP1, and OTX2 in the periphery. This labeling is reduced or undetectable upon exposure to higher concentrations of CHIR (6/9/12 μM). **d** *N* = 14 organoids (3 μM CHIR), 9 organoids (6 μM CHIR), 5 organoids (9 μM CHIR treated), and 14 organoids (12 μM CHIR treated) from 2 independent experiments were used for analysis (each organoid was considered as an independent biological replicate). Additional examples are provided in Supplementary Fig. S10. **e** q-PCR analysis shows that the expression of the ChP markers *TTR, AQP1*, and *OTX2* is upregulated in organoids at day 30 organoids exposed to 3 μM CHIR from day 18. This increased expression is lost upon treatment with 12 μM CHIR for the same period. (**f**) *LEF1* expression displays a stepwise increase upon 3 μM and 12 μM CHIR treatment, consistent with increased activation of the canonical Wnt pathway. Bar graphs (**e**, **f**) represents mean ± SEM. Group means were compared using one-way ANOVA followed by post hoc Tukey’s multiple comparison test, *p* < 0.0001(**e**, **f**), *p value<0.05, **p value<0.01, ***p value<0.001; (**e**, **f**) *N* = 8 biologically independent replicates examined over 3 independent experiments. (**g**) Heatmap and hierarchical clustering comparing normalized RNA-seq reads for 3 μM and 12 μM CHIR-treated organoids. Several ChP-enriched genes are downregulated with increased activation (12 μM CHIR) of canonical Wnt signaling (*N* = 3 biologically independent replicates). Scale bars: 100 μm (all panels in **c** and **d**). Source data are provided as a Source Data file.

We examined the effect of increased activation of canonical Wnt signaling in the hESC-derived organoids. We tested a range of concentrations of CHIR (3–12 μM) in the cocktail added to the organoids after 18 days in vitro, keeping the BMP4 concentration constant. The organoids were examined for the presence of *Ttr/* TTR, *Aqp1*/ AQP1, and *Otx2*/ OTX2 by immunohistochemistry, qPCR analysis, and RNA-Seq (Fig. 10). As early as 7 days post CHIR treatment (25 days in vitro), 3 μM-treated organoids displayed multiple domains of TTR decorating the perimeter, but 12 μM-treated organoids displayed no detectable TTR (Fig. 10c; Supplementary Fig. 10c). We tested the dose-response to CHIR treatment by immunostaining for TTR, AQP1, and OTX2 in organoids using the different CHIR regimens. Whereas all three proteins appeared along the perimeter of 3 μM CHIR-treated organoids, they were undetectable in organoids treated with higher CHIR concentrations (Fig. 10d; Supplementary Fig. S10d). qPCR analysis confirmed this finding: upon treatment with 3 μM CHIR, *TTR, AQP1*, and *OTX2* displayed a significant increase in fold expression over the baseline (untreated 18-day organoids). However, treatment with 12 μM CHIR appeared to suppress the expression of these genes to levels below the baseline (Fig. 10e). In contrast, *LEF1* expression correctly reflected the increased activation of canonical Wnt signaling by displaying a graded increase in fold expression above baseline upon 3 μM and 12 μM CHIR treatment (Fig. 10f).

To obtain a comprehensive picture of the effect of increased canonical Wnt pathway activation on hESC-derived organoids, we performed RNA-seq of 3 μM and 12 μM CHIR-treated samples. Several major ChPe markers detected in the 3 μM-treated condition are suppressed upon 12 μM CHIR treatment (Fig. 10g). These include markers of differentiated ChPe such as *AQP1, TTR, OTX2*; genes that encode proteins that participate in forming tight junctions that are typical of the ChPe, such as *CLDN1, 2, 5*, and *TJP1,3*; and those that encode transporters or ion channels such as *CLIC6, SLC4A10, SLC12A7, and SLC12A8*. The transcriptome of the 12 μM CHIR-treated organoids did not display upregulation of neuronal markers. One possible reason for this could be that any such transformation would have been limited to the periphery of the organoid, and the corresponding transcriptomic changes may have become masked due to the larger contribution of the rest of the organoid. We, therefore, examined 3, 6, 9, and 12 μM CHIR-treated organoid samples by immunohistochemistry using a neuronal marker that is enriched in the hippocampus, ZBTB20^42^ and a choroid epithelium marker OTX2. We discovered that with increasing CHIR treatment, the organoid samples reveal a significant reduction in OTX2 + cells and a corresponding increase in ZBTB20 + cells (Supplementary Fig. S11). Additional analysis would be needed to test whether this indeed represents a transformation to a neuronal identity.

In summary, stabilizing β-CATENIN in hESC-derived organoids leads to the loss of ChPe identity, consistent with the findings in the β-Catenin GOF mouse.

## Discussion

The ChP actively processes canonical Wnt signaling from the earliest stages of its development. Expression of the reporter BAT-Gal and target gene *Axin2* was reported in the choroid plaque at E10.5 and the ChPe at E11.5^34^. Similarly, the expression of both *Lef1*, as well as a LEF1-βGal allele, were reported in the E12.5 mouse ChP^43^. Furthermore, the expression of *Lef1, Axin2*^43,44^, *Tcf7* (named *Tcf1* in^45^), several Frizzled receptors (*Fzd1, Fzd2, Fzd3*, and *Fzd7*)^46^ as well as the phosphorylated LRP6 co-receptor is present in the developing ChPe^47^. Consistent with these reports, we found that loss of β-CATENIN, which mediates canonical Wnt signaling, disrupts the normal development of the telencephalic ChP.

It appears, however, that a regulated level of canonical Wnt signaling is critical for ChPe differentiation. Constitutive activation of canonical Wnt signaling results in a profoundly defective ChPe both in the mouse embryo and in hESC-derived ChP organoids. Since this pathway regulates multiple aspects of cellular, tissue, and organ development, several other systems that require canonical Wnt signaling for normal development also display an array of defects upon its constitutive activation. During human hair follicle morphogenesis, loss or constitutive activation of β-CATENIN leads to tumor formation or defective differentiation, respectively^48,49^. There are some examples in which constitutive activation of β-CATENIN appears to transform cell identity. The mouse epiblast prematurely displays epithelial to mesenchymal transition^50^; endothelial cells of the midbrain transform to a “barrier cell” identity^51^, and airway epithelial cells take on a neuroendocrine-like fate^52^. Our study reports an apparent transformation of specified ChPe cells to a neuronal fate upon constitutive activation of β-CATENIN in their progenitor domain, the hem, which parallels the findings in the other systems mentioned above.

An unexpected feature is that the transcriptome of the β-catenin GOF ChP has some components that fit with a CR cell-like profile, such as *Ebf2*, *Jph3, Reelin*, *Slit1*^53^, and others that fit with a medial cortical/hippocampal-like profile *(Lef1, Prox1, Wif1)*, in addition to an array of cortical neuroepithelial or postmitotic neuronal markers (Supplementary Table 1). While it is not possible to delineate whether there is a heterogenous population of CR-like, hippocampal-like, and cortical neuron/neuronal progenitor-like cells in the β-catenin GOF ChP using bulk RNAseq, it appears that the overall picture is one of the mixed transcriptomic signatures, at least at a population level. Fate choice between ChPe and CR cells is regulated by transcription repressors of the bHLH family members, HES1, 2, and 3, that promote epithelial identity by suppressing neurogenic genes and inducing BMP signaling^54^. Upon loss of all three *Hes* genes, the hem upregulates the pro-neuronal factor NEUROG2 and produces CR cells instead of ChPe cells^55^. Our results suggest that the canonical Wnt pathway may intersect with this regulatory mechanism. β-CATENIN is also involved in cell adhesion, which is an important factor in the epithelial organization. In addition, the hem also expresses *Wnt5a*^12^ and WNT5a acts via the non-canonical Wnt-PCP pathway to regulate ChPe cytoarchitecture, apicobasal polarity, and overall size and complexity of the ChP^18^. BMPs are known to be critical for the specification of the ChP^16^. Thus, multiple signaling molecules of the WNT and BMP families present in the hem are each critical for ChP development. A comprehensive analysis of interactions between these factors and the pathways they regulate may offer insight on the regulation of multipotency of progenitors in the hem, and the temporal trajectory of ChPe differentiation.

Activation of the canonical Wnt pathway is often tumorigenic^21^. Human cases of ChP carcinoma are associated with mutations in exon 3 of the *ß-CATENIN* gene that lead to its constitutive activation^20,21^. Mouse models of ChP papilloma that are generated by constitutive activation of NOTCH signaling show concomitant upregulation of the canonical Wnt pathway^22^. It is not clear what mechanisms control whether canonical Wnt activation causes cell fate transformations or tumorigenic changes, but it is likely that crosstalk with other major signaling pathways is involved. The β-catenin GOF mouse ChP offers a useful and accessible model in which this question can be examined.

Key features of β-catenin GOF in the ChP include dysregulation of factors that are essential for the ChP to produce the CSF. These include several anion cation exchangers, ion channels, and solute transporters, each of which acts as a regulator of CSF composition: a sodium-potassium ATPase, *Atp1a1*^55^; an epithelial sodium channel, *Scnn1a*^56^; an inward rectifying potassium channel, *Kcnj13*^57^; a voltage-gated potassium channel, *Kcna1*^58^; and a sodium-potassium-chloride cotransporter, *Nkcc1*, that modulates water transport underlying CSF production^59^ and is also associated with Blood CSF barrier disruption^60^. Furthermore, the expression of several genes encoding for glucose, chloride, and amino acid transporters, is also downregulated in the β-catenin GOF ChP. Together, the data suggest that canonical Wnt signaling may be an important factor in enabling the ChPe to produce normal CSF. Mutations in exon 3 of *ß-CATENIN* in humans result in stabilization of β-CATENIN^61^, similar to that seen in the β-catenin GOF mouse model^26^. Human studies have focused on the association of these mutations with carcinomas^21,61^, but no associated effects on the CSF have been explored. Our study identifies a potentially important developmental outcome of the β-catenin GOF perturbation in terms of its possible effect on the composition of the CSF. The formation of ChPe in hESC-derived organoids requires low activation of the canonical Wnt pathway together with BMP signaling. Loss of ChPe fate in these organoids upon high activation of the canonical Wnt pathway indicates conservation of the role of this pathway in the development of the ChPe. hESC-derived organoids are not only an excellent system to study conserved developmental mechanisms but also offer the opportunity to test for subtle effects of altered ChP/CSF status on neuronal cells. Depending on the protocol used, the organoids develop into fluid-filled cysts that contain several human CSF proteins^62^. These organoids are permeable to small molecules and are excellent model systems to test modalities of drug delivery to the CNS^62^. Thus, hESC-derived ChP organoids offer a suitable system to model both diseases, as well as modalities for treatment.

In conclusion, we demonstrate that the developing choroid plexus expresses components of the canonical Wnt signaling pathway in embryonic mouse and human brains. Both loss and gain of function of β-CATENIN disrupt ChPe development, indicating that this pathway is likely to be under a finely tuned regulation in vivo. These findings motivate future studies on how this regulation is achieved and may provide a mechanistic basis for subtle deficits in ChP/CSF function connected to the disruptions of the canonical Wnt pathway function in this system.

## Methods

### Tissue collection

#### Mice

All animal protocols were approved by the Institutional Animal Ethics Committee of the Tata Institute of Fundamental Research, Mumbai, India. The mouse lines used in this study were kind gifts from Kathy Millen (Center for Integrative Brain Research, Seattle Children’s Research Institute; Lmx1aCre line^25^), Raj Awatramani (Department of Neurology and Center for Genetic Medicine, Northwestern University Feinberg Medical School Chicago, Illinois; β-catenin LOF mice^31^, Michael J. Holtzman (University of Washington, St. Louis), USA; Foxj1Cre line^36,37^. The Ai9 reporter mouse line was obtained from JAX labs Stock No. 007909. All animals were kept in an ambient temperature and humidity, with a 12 hr light-dark cycle and food available ad libitum. Noon of the day of the vaginal plug was designated as embryonic day 0.5 (E0.5). Animals of both sexes were used for generating the control and Foxj1Cre::β-catenin GOF brain samples. For Lmx1aCre animals, since the transgene is on the X-Chromosome, females showed mosaic expression of the Cre recombinase due to random X inactivation. Cre-positive male embryos/pups were used for analysis except from Fig. 6 and Supplementary Fig. S7 where female embryos were analyzed. Controls were littermates wherever possible. Control embryos/pups carrying the Ai9 reporter to mark the Lmx1a or Foxj1 lineage carried the respective Cre transgenes. Breeding colonies were maintained with the female breeders carrying the Lmx1aCre and Foxj1Cre transgenes since this germline recombination occurs in the testes of these lines. Primers used for genotyping were: Cre F: 5′ATTTGCCTGCATTACCGGTC3′, Cre R: 5′ATCAACGTTTTCTTTTCGG3′, Cre-positive DNA shows a band at 350 bp. For detecting β-catenin LOF (Exon2–6 floxed) following primers were used: RM41 5′AAGGTAGAG TGATGAAAGTTGTT3′; RM42 5′CACCATGTCCTCTGTCTATTC3′; RM43 5′TACACTATTGAATCACAGGGACTT3′^31^. The PCR shows 221 bp as Wild type band while 324 bp as band corresponding to floxed allele. For genotyping β-catenin GOF (Exon 3 floxed) FP 5′ GCTGCGTGGACAATGGCTAC3′ and RP 5′GCTTTTCTGTCCGGCTCCAT3′^26^ was used which detects conditional allele at 550 bp and wild type band at 350 bp in PCR.

#### Human

The Institutional Ethical Review Board of the School of Medicine, University of Zagreb, and University Hospital Center Zagreb have approved the procedure for collecting postmortem brain samples and their use for research purposes in the study under protocol numbers: UHC Zagreb EP02/21AG; Class 8.1–17/170-2 and UZSM: 641-01/19-02/01, No. 380-59-10106-19-111/201). Fetal brain specimens were obtained in autopsies following elective termination or spontaneous abortions with the written informed consent of the mother, according to the Helsinki declaration (compensation to mothers was not involved). The fetal age was estimated based on crown-rump length (CRL, in mm), pregnancy records, and histological findings and expressed as GW. In addition, the correlation of maturational parameters (CRL, body mass, pregnancy records, and sonographic examination) revealed no evidence of growth retardation or malformations. Finally, specimens in which subsequent postmortem analysis revealed macroscopically or microscopically identified pathological changes, or postmortem autolytic changes, were excluded.

### Choroid plexus dissection

All dissections were performed in ice-cold PBS. For wholemount immunostained ChP preparations, telencephalic hemispheres were isolated using #5 forceps under a stereo zoom microscope (Nikon SMZ445). The rostral part of the ChP was gently extracted from the telencephalic ventricle using blunt forceps. The ChP was dissected using microdissection scissors while stabilizing the hemisphere using blunt forceps. For RNA Seq/ qPCR experiments, we used a line carrying the Ai9 reporter to avoid contamination from adjacent tissues. We examined the brains for Ai9 fluorescence before and after microdissection to ascertain that the Ai9-positive hem remained intact (Fig. 2d). Furthermore, we isolated only the region of the ChP that was easily accessible and protruded into the telencephalic ventricle.

### Immunohistochemistry

#### Mouse sections and organoids

Mouse brain sections/organoid sections were mounted on plus slides (Catalogue number: EMS 71869-11) and dried for 2–3 h. Slides were transferred to a slide mailer (Catalogue number: EMS 71549-08) containing PBS + 0.01% TritonX-100 for 10 min followed by a wash with PBS + 0.03% TritonX-100 for 5 min. For antigen retrieval, sections were boiled in a 10 mM sodium citrate buffer (pH = 6) at 90°C for 10 min using a water bath. Slides were cooled to room temperature and washed with PBS + 0.01% TritonX-100 for 10 min. Blocking (5% horse serum/Lamb serum in PBS + 0.1% TritonX-100) for 1 h followed by overnight primary antibody incubation at 4 °C. Secondary antibody incubation was performed at room temperature for 2 h followed by three washes with 1x PBS. Slides were mounted using vectashield mounting media (Vector laboratories H-1000-10) and imaged in an Olympus FluoView 1200 confocal microscope. The primary antibodies used were: LEF1 (rabbit, 1:200 CST catalogue # C12A5), β-CATENIN (mouse 1:200, BDbiosciences catalogue # 610153), β-CATENIN (rabbit 1:50, CST catalogue # 8814), RFP (rabbit, 1:200 Abcam catalogue # ab62341), FZD1 (rabbit, 1:100 catalogue # LS-A4150), AXIN2 (rabbit, 1:200 Abcam catalogue # ab185821), TTR (rabbit 1:100, DAKO catalogue # A0002), AQP1 (mouse, 1:200 SCBT catalogue # sc25287), OTX2(mouse, 1:200 SCBT catalogue # sc-514195), OTX2(1:500,invitrogen,PA5-39887), AQP1 (rabbit, 1:200,invitrogen catalogue # MA5-32593), E-CADHERIN (mouse, 1:200, SCBTcatalogue# sc21791), E-CADHERIN (mouse,1:100 BD Bio transductions, catalogue # 610182 0Cleaved CASPASE 3 (rabbit, 1:200 CST catalogue # 9664), Phospho HISTONE (rabbit, 1:200 CST catalogue # H0412), TBR2 (rabbit, 1:200 Abcam catalogue # ab23345), PAX6 (rabbit, 1:500 Abcam catalogue # ab195045), PROX1 (rabbit, 1:500 Millipore catalogue # ab5475), β-III TUBULIN (mouse, 1:100 Promega catalogue # G7128), BLBP (rabbit, 1:200 sigma catalogue # ABN14), ACTIN (mouse, 1:500 sigma catalogue # A2228), LAMININ (rabbit, 1:200 sigma catalogue # L9393). Secondary antibodies used were following: Goat Anti Rabbit Alexa fluor 488 (1:200, Invitrogen catalogue # A11034), Goat Antimouse Alexa fluor 594 (1:200, Invitrogen catalogue # R37121), Goat Anti Rabbit Alexa fluor 568 (1:200, Invitrogen catalogue # A11011), Donkey Anti goat Alexa fluor 568 (1:200, Invitrogen catalogue # A32814), Donkey Antirabbit Alexa fluor 647 (1:200, Invitrogen catalogue # A31573).

#### Human

The entire brains were fixed by immersion in 4% paraformaldehyde in 0.1 M phosphate buffer saline (pH = 7.4). Subsequently, tissue blocks were embedded in paraffin wax. Serial sections were cut (5 μm) and stained. The cresyl violet staining was used to delineate cytoarchitectonic boundaries, while adjacent sections were labeled using immunofluorescence with various antibodies. The following primary antibodies were used: (1) rabbit polyclonal anti-Axin 2, (ab185821, Abcam); (2) the rabbit polyclonal Frizzled 1 antibody, FZD1 (LS-A4150, LSBio); (3) the monoclonal rabbit LEF1 antibody (C12A5, CST); (4) the mouse monoclonal aquaporin1 antibody, AQP1 (sc-25287, SCBT); (5) Rabbit nonphospho (Active) βCatenin (D13A1, Cell Signaling, Leiden, Netherlands); (6) monoclonal mouse OTX2 antibody (MA5-15854, Invitrogen); (7) the mouse monoclonal Beta-Catenin, (M3539, Dako Agilent). The dewaxing of sections was performed in xylol for 5 min and a series of alcohol solutions (100%, 96%, 70%) for 2 min. Slides were then washed in PBS with 0.03% triton (10 min). The antigen retrieval was performed in citrate buffer pH6.0 and microwave (800 W) for 10 min. The slides were cooled at room temperature and immersed in a blocking solution (PBS containing 3% bovine serum albumin, BSA, and 0.01 % Triton X-100, all from Sigma, St. Louis, MO) for 30 min. to prevent nonspecific background staining. Sections were then incubated with primary antibodies for 48 h at 4 ˚C (AXIN2 1:100, FZD1 1:100, LEF1 1:100, AQP1 1:100, OTX2 1:50, β-Catenin cell signaling 1:400, β-Catenin Dako 1:200), washed and subsequently incubated with secondary antimouse or antirabbit antibodies diluted in PBS (at 1:1000) for 2 h at the room temperature. For visualization of the specific immune reactivity following secondary antibodies were used according to the laboratory protocol^63^: the donkey antimouse IgG Alexa fluor-plus 488, donkey antirabbit IgG Alexa fluor-plus 647, donkey antirabbit IgG 488, donkey antimouse IgG Alexa fluor 546 were used. Sections were covered with a mounting medium containing DAPI. Negative controls were included in all experiments by replacing the primary antibody with a blocking solution. No immunolabeling was detected in the control sections.

### In situ hybridization

Plasmids used to generate probes for in situ hybridization were kind gifts from Elizabeth Grove (*Axin2, Wnt3a, Ttr*), Cliff Ragsdale (*Fzd1, Fzd2a*) Kathleen Millen (*Lmx1a*). Fixed mouse brains were sectioned using a freezing microtome (Leica SM2000R Sliding Microtome) at a thickness of 20 μM. Sections were mounted on super frost plus slides (Catalogue #: EMS 71869-11), postfixed with 4% (w/v) paraformaldehyde for 15 min, washed three times with PBS. The sections were then treated with proteinase K dissolved in Tris-EDTA buffer (1 μg/ml). Post fixation was performed in 4% PFA for 15 min, and the sections were washed three times in PBS. Hybridization was performed for 16 h at 70 °C in a buffer containing 50% (v/v) formamide, 2X SSC, and 1% (w/v) SDS. Digoxigenin (DIG)-labeled cRNA probes were used for hybridization and were prepared from the respective plasmids using the in-vitro transcription. After hybridization, three stringent washes (for 45 min each) were performed in solution X (50% formamide, 2X SSC, and 1% SDS) followed by a wash with 2XSSC and then 0.2XSSC. Blocking was performed for 1 hr with 10% horse serum in TBST (Tris-buffered saline pH 7.5 with 0.1% Tween-20). Sections were incubated in alkaline phosphatase-conjugated anti-DIG (Digoxygenin) antibody Fab fragments (1:5000; Roche, catalog #12486523) at 1:5000 in the blocking buffer and incubated at 4 °C overnight. The color reaction was performed using NBT/BCIP substrate (Roche, 4-nitroblue tetrazolium chloride, catalog #70210625; 5-Bromo- 4-chloro-3-indolyl phosphate, catalog #70251721). Counterstaining was performed using Fast Red (Sigma-Aldrich, catalog # N3020). Slides were dried and coverslipped using DPX mounting reagent (SDFCL, catalog #46029). Probes used for in-situ hybridization were generated using an in-vitro transcription reaction. Linearized templates for this process were generated by restriction digestion of plasmids (described in^64^). Plasmids used to generate probes for in situ hybridization were kind gifts from Elizabeth Grove (*Axin2, Wnt3a, Wnt2b, Ttr*), Cliff Ragsdale (*Fzd1, Fzd2a*) Kathleen Millen (*Lmx1a*).

### Image acquisition and analysis

Bright-field images were acquired using Zeiss Axioskop-2 plus microscope equipped with a Nikon DS-fi2 camera and associated software (NIS Elements V4.0). Mouse sections were imaged in Olympus FluoView 1200 confocal microscope and the human brain sections were imaged in Olympus FluoView 3000 confocal microscope. All the organoid sections were imaged using the Andor Dragonfly spinning disk confocal microscope system. All the image analysis was done on Fiji-ImageJ, Imaris (Version 7.2.3), and/or Adobe Photoshop CS6.

For intensity quantification (Fig. 1e), a line was drawn across the cell using the “line tool” option in Fiji (arrowheads mark the origin), and “grey values” were measured across the line.

For area measurements (Fig. 3b) ROIs were drawn using the “freehand selection tool” around the *Ttr* + domains, and area was calculated. In the Lmx1a Cre:: β Catenin GOF brains where the *Ttr* + domains appear to be sparse, multiple ROIs were drawn separately and added in the ROI manager. The area for each *Ttr* + domain was calculated and summed to obtain the total area.

For nuclear intensity quantification of β-Catenin GOF (Fig. 1j; Fig. 2c and i; Supplementary Fig. S2b and d; Fig. 3d; Fig. 5e) control and Lmx1a Cre::β Catenin GOF/LOF coronal sections were mounted on the same slide and processed for immunostaining. Images were acquired under similar PMT settings with a pixel distribution of 1024 ×1024 in “sequential scan mode”. The PMT setting was optimized such that saturation can be avoided. The z stack images are projected as “SUM slices”and bit depth was set as “8 bit”. From a composite image containing DAPI and markers, ROIs were drawn around the DAPI + nuclei using the “freehand selection tool” in Fiji. When drawing the ROIs, the channel having markers was hidden using the “channel tool” to avoid any visual bias. The nucleus for which the boundary is not clear (overlapping with each other) was not scored. After completion of marking the ROIs using the DAPI channel, the marker channel was turned on and from this channel, “mean grey values” and “area” were measured using the “measure” option in the ROI manager. Our results are consistent with a recent study that examined the subcellular localization of β-Catenin and reported a substantial cytoplasmic component even when the nuclear accumulation is increased upon Wnt activation^65^.

For cell counts (Fig. 3e; Fig. 4c and e; Supplementary Fig. S4b, c, and d; Fig. 5d) “cell counter” plugin in Fiji was used. Cells for which the boundary was not adequately resolved were excluded. While scoring for DAPI + or AI9 + cells, the channel containing the marker was hidden using the “channel tool” to avoid any visual bias. Similarly, while scoring markers, the Ai9 or DAPI channel was hidden using the “channel tool”. Similarly, ROI was drawn around the cytoplasm using the β-III TUB channel in Fig. 6 and intensity was quantified from the Alexa 488 (TTR) and Alexa 647 (β-III TUBULIN) channels.

For Supplementary Fig. S11c and d, a region of width 100 µm was drawn using a “freehand line tool,” and the marker+ cells were scored using the “cell counter” plugin in Fiji.

Image stitching was performed for Fig. 4d and f (low mags) using the “pairwise stitching” plugin in Fiji. The “subpixel accuracy” parameter was selected for all the stitching operations.

Nonlinear operations e.g. gamma adjustment were not performed in any of the figures. Brightness and contrast adjustments were performed identically for control and mutant conditions.

### Generation of hESC-derived organoids

The human ES cell line WIBR3, obtained from the Whitehead Institute for Biomedical Research, (https://hpscreg.eu/cell-line/WIBRe001-A), was used in compliance with all applicable statutes and regulations and compliance with applicable guidelines (including the NAS Guidelines or guidelines of the International Society for Stem Cell Research as relevant to the conduct of human embryonic stem cell research [“ISSCR Guidelines”]. The line was cultured on an irradiated MEF (mouse embryonic fibroblast) feeder layer. To maintain the pluripotent state, a customized human Naïve media with 40 μM Rock Inhibitor Y-27632 was used as described in Karzbrun et al., 2018^66^. Differentiation of hES aggregates into Choroid plexus-like organoids was done using a protocol described in Sakaguchi et al. 2015^39^. Briefly, hESC colonies were grown in a Matrigel-coated plate till confluence. The cells were dissociated with trypsin and rapidly reaggregated in an ultra-low attachment 96 well V bottom plate (9000 cells per well) containing a media termed SA1 media. This media contains DMEM (Invitrogen cat # 11965092) supplemented with 20% (vol/vol) KSR (Invitrogen cat # 10828028), 0.1 mM non-essential amino acids (Invitrogen cat # 11140050), 1 mM pyruvate (Invitrogen cat # 11360070), 0.1 mM 2-mercaptoethanol (Invitrogen cat # 21985023), 100U/ml penicillin and 100 mg/ml streptomycin. 0.3 mM IWR1e (tankyrase inhibitor, MedChemExpress cat# HY-12238) and 5 mM SB431542 (TGFβ inhibitor; MedChemExpress HY-10431) were added to culture from day 0 to day 18 (day of aggregation was defined as day 0). The culture media used after day 18 till day 40 was termed as SA2, which contains DMEM/F-12 (Invitrogen cat # 21331020), GlutaMAX(TM) (Gibco) supplemented with 1% N-2 supplement (Invitrogen cat # 17502048), 1% Chemically Defined Lipid Concentrate (Invitrogen cat # 11905031), fetal bovine serum (FBS; 10% vol/vol), 3 μM CHIR 99021 (GSK3 inhibitor; MedChemExpress cat # HY-10182) and 0.5 nM BMP4 (R&D cat # 314-BP-050) under normoxia condition. The medium was changed once every 2 days. From day 5 onwards, the plates were kept on an orbital shaker placed inside the incubator. On day 18 the organoids were transferred from 96 well plates to a 10 cm ultra-low attachment plate (6 organoids per plate) and kept on an orbital shaker inside the incubator. For sample collection, the organoids were rinsed with 1x PBS 2 times, then either flash-frozen for RNA-seq experiments or transferred to a small dish containing 4% PFA solution for immunohistochemistry.

### RNA sequencing, library preparation, and analysis of mouse tissues

The mouse ChP was dissected from E14.5 control and β-catenin GOF brains and stored in Trizol^Ⓡ^ reagent. Extracted RNA of 1 μg (RIN > 7.5, measured using the Agilent 2100 bioanalyzer) from ChPe dissected from 4 embryos was pooled for each of two biological replicates. After library preparation, sequencing was performed on the Illumina platform to achieve 100 bp or 150 bp reads to generate 30 Million paired-end reads per sample. FastQ QC was performed as described in^67^, and reads > 30 Phred scores were aligned using HISAT2^68^. Feature counts were used to quantify the number of reads per transcript. Differential expression analysis was performed using DESeq2 v3.14^69^ on the R platform (v3.6.3). Genes showing |log2 Fold change | ≥1 were used for further analysis. Gene ontology analysis was performed using geneontology.org or gprofiler.org. Semantics were summarized using “REVIGO”^70^, and bar plots were created with GraphPad Prism V9.1.0. Heat maps and sample correlation plots were plotted using R studio. Gene-based heatmaps were plotted using normalized reads on Morpheus (Morpheus, https://software.broadinstitute.org/morpheus).

### RNA sequencing, library preparation, and analysis of organoids

Organoids generated in Figs. 9 and 10 were pooled such that for one biological replicate, 3 organoids were used, and three such biological replicates were performed for day 18 and day 30 organoids. Bulk MARS-seq libraries were produced from 50 ng of total RNA as previously described^71^. Libraries were then sequenced with 75 bp single-end read on Illumina Nextseq500 platform, and FASTQ files were processed and analyzed using UTAP^72^. Briefly, sequenced reads were trimmed using cutadapt (parameters: -a adaptor -a “A {10}” –times 2 -u 3 -u -3 -q 20 -m 25) and were mapped to hg38 indexed reference genome using STAR^73^ v2.4.2a with the following parameters: –alignEndsType EndToEnd, –out Filter Mismatch Nover Lmax 0.05, –two pass Mode Basic). UMIs were counted after marking duplicates using HTSeq-count in union mode. For the number of reads per gene, 1000 bp of 3′ end Gencode annotated transcripts were counted. The total RNA-seq from 3 μM and 12 μM CHIR-treated organoids (Fig. 10) were done using TruSeq stranded mRNA library kit and sequenced by DNA Link Sequencing Lab, Korea, on NovaSeq 6000, 100PE. Data were analyzed with the UTAP pipeline^71^, where adapter trimming and reference genome alignment were performed as described above. Read count per gene was done with STAR. Normalization of the counts and differential expression analysis was performed using DESeq2 (v3.14)^69^ with the parameters: betaPrior=True, cooks Cutoff=FALSE, independentFiltering=FALSE. Raw P values were adjusted for multiple testing using the procedure of Benjamini and Hochberg. Genes with log2FC > 1 or <−1, *p* adjust < 0.05 and baseMean > 10 were considered as differentially expressed. GO Biological process overrepresentation test on differentially expressed genes between different time points of organoid growth was performed with the package clusterProfiler^74^.

### qPCR Analysis

#### Mouse

ChP samples from control and Lmx1aCre::β-catenin GOF E14.5 embryos were collected in a 1.5 ml tube with 200 μl of Trizol reagent. ChP from 2 brains were pooled together and considered 1 biological replicate and 3 such biological replicates were performed. Total RNA was extracted using Trizol reagent following the manufacturer’s protocol. RNA concentration was measured using RNA-HS assay kit (catalog # Q32852) in QBIT-2 fluorometer (catalog # Q32866). cDNA was synthesized using SuperScript™ IV kit (catalog # 18091050). Real-Time qPCR reactions were performed in triplicates using KAPA SYBR FAST qPCR Kit (2X) (catalog # KK4601) on LightCycler^®^ 96 Real-time PCR system following the manufacturer’s recommendation. For each primer, the annealing temperature and primer concentration was optimized using gradient PCR. Melt curve analysis was performed to rule out the possibility of nonspecific amplification. GAPDH was used as a reference gene, and analysis was performed using the ΔΔ threshold cycle (Ct) method. The fold changes were represented as mean ± SEM.

#### Primers

*Axin2*, FP: 5′-GAAGAAATTCCATACAGGAGGAT-3′; RP: 5′-GTCACTCGCCTTCTTGAAATAA-3; *Lef1*, FP: 5′-AGAAATGAGAGCGAATGTCGTAG-3′; RP: 5′-CTTTGCACGTTGGGAAGGA-3′; *Aqp1*, FP: 5′-CTGCTGGCGATTGACTACACTG-3′; RP: 5′-GGTTTGAGAAGTTGCGGGTGAG-3′; *Otx2*, FP: 5′-CAAAGTGAGACCTGCCAAAAAGA-3′; RP: 5′-TGGACAAGGGATCTGACAGTG-3′; *Slc12a2*, FP: 5′-GCAAGACTCCAACTCAGCCAC-3′; RP: 5′-ACCTCCATCATCAAAAAGCCACC-3′; *Slc12a4*, FP: 5′-GCCCCAACCTTACTGCTGAC-3′; RP: 5′-TCTCCTTTAGGCCGAGGGTG -3′; *Slc4a10*, FP: 5′-TTCAAGACCAGCCGCTATTT-3′; RP: 5′-GGATCCCAATGGCATAGTCA-3′; *Slc4a2*, FP: 5′-TCCAGAGCGAGCGGGTTATG-3′, RP: 5′-GAGGACTGGCGGTGGTACTCAAAGTC-3′; *Ttr*, FP: 5′-CCGTGTTAGCAGCTCAGGAA-3′; RP: 5′-GGGTTTTAGGAGCAGGGGAG-3′; *Gapdh*, FP: 5′-ATTCAACGGCACAGTCAAGG -3′; 5′-TGGATGCAGGGATGATGTTC-3′.

#### Organoids

Total RNA of Day 18 and Day 30 organoids was extracted using the RNeasy Mini kit (Qiagen, Germany) following the manufacturer’s protocol and followed by DNAse I treatment. RNA concentration was measured using Nanodrop (Thermo Scientific, MA, USA). cDNA was synthesized using M-MLV reverse transcriptase (M3682, Promega, Wisconsin, USA). Real-Time reactions were performed in triplicates using KAPA SYBR FAST qPCR Kit (2X) on QuantStudio 5 Real-time PCR system (Bio-Rad, CA, USA) following the manufacturer’s recommendation. Expression levels were normalized against GAPDH using the ΔΔ threshold cycle (Ct) method. The fold changes were represented as mean ± SEM. Primers: *AQP1*, FP: 5′-TGGACACCTCCTGGCTATTG-3′; RP: 5′-GGGCCAGGATGAAGTCGTAG-3′; *LEF1*, FP: 5′-AGAACACCCCGATGACGGA-3′; RP: 5′-GAGGGTCCCTTGTTGTAGAGG-3′; *TTR*, FP: 5′ ATCCAAGTGTCCTCTGATGGT 3′; RP: 5′ GCCAAGTGCCTTCCAGTAAGA 3′; *OTX2*, FP: 5′-AGAGGACGACGTTCACTCG-3′; RP: 5′-TCGGGCAAGTTGATTTTCAGT-3′.

### Statistics and reproducibility

Biological replicates (*n*) denote samples obtained from individual brains/ organoids. The total numbers of embryos/ brains analyzed for immunohistochemistry or in situ hybridization for each genotype were: 69 Control brains (Figs. 1–6),10 Lmx1aCre::β-catenin LOF brains (Supplementary Fig. S2 and 3), 46 Lmx1aCre::β-catenin GOF brains (Figs. 2,3,4,6 and 7; Supplementary Fig. S3,4,5,6 and 7), 7 Foxj1Cre::β-catenin GOF brains (Fig. 5 and Supplementary Fig. S5). Detailed information about biological replicates and sample size were described in the corresponding figure legends. For Fig. 1a–g Representative images are shown from different sections taken from 1 fetal brain at each age (GW11 & 13). For Fig. 4f–I; 6h–m representative images are shown from 3 biologically independent replicates and the quantification is shown from one biological replicate for best representation purposes. For qPCR and RNAseq, each biological replicate consisted of samples pooled from more than one brains that were processed as one sample. For qPCR (Figs. 2 and 3), samples from 2 brains were pooled, while for RNA Seq (Figs. 2 and 7, 8 Supplementary Fig. S8), samples from 4 brains were pooled and considered one biological replicate. For organoids, one organoid was an independent biological replicate. A total of 48 control organoids (3 μM CHIR) were used for Fig. 9. For Fig. 10 and Supplementary Fig. S10, 14 organoids (3 μM CHIR), 9 organoids (6 μM CHIR), 5 organoids (9 μM CHIR-treated), and 14 organoids (12 μM CHIR treated), were used for immunohistochemistry analysis. For RNA-Seq experiments (Figs. 9 & 10), 3 organoids (per treatment condition) are pooled as one biological replicate. All replicates were successful and no data were excluded. The mouse genotypes could be distinguished by apparent phenotypic features, so it was not possible to perform the quantifications in a blinded manner. During image analysis, stringent measures were followed to avoid bias as described in the “Image analysis” section. All statistical analysis was performed in Graph Pad Prism (V7.0 & V9.1.0), the exact test and “P-value” information were provided in corresponding figure legends and Source data file. For all statistical tests confidence interval is always 95% (α = 0.05). For all the bar graphs and scatterplots error bars indicate “standard error of mean”. For all violin plots Solid black line represents median and dotted lines represents quartiles.

### Reporting Summary

Further information on research design is available in the Nature Research Reporting Summary linked to this article.

## Supplementary information

Supplementary Information

Description of Additional Supplementary Files

Supplementary data 1

Reporting Summary

## Data Availability

The mouse RNA-seq data (Figs. 2,7,8, Supplementary Figure S1& 8) generated in this study have been deposited in the GEO database under accession code: “GSE162784”. The organoid MARS-seq and RNA-seq datasets (Figs. 9,10, Supplementary Fig. S9) have been deposited in the GEO database under accession code “GSE162808”. All other relevant data supporting the key findings of this study are available within the article and its Supplementary Information files or from the corresponding author upon reasonable request. Source data are provided with this paper.

## Source data

Source Data

## Code availability

All the codes used for analysis were published previously (cited and mentioned in details in “methods” section). All codes are available on request with the corresponding author. The source code for UTAP software used in this manuscript for the analysis of MARS-seq and RNA-seq data (Fig. 9, 10) is available at https://bitbucket.org/bbcu/utap.
