# Supplementary material for: Constitutive activation of canonical Wnt signaling disrupts choroid plexus epithelial fate
Source: Nat Commun. 2022 Feb 2;13:633. doi: 10.1038/s41467-021-27602-z (PMC8810795; doi:10.1038/s41467-021-27602-z)
Supplement: Supplementary file 1 — Supplementary Information [file 41467_2021_27602_MOESM1_ESM.pdf]

Constitutive activation of canonical Wnt signaling disrupts choroid plexus epithelial fate

Supplementary Information

Arpan Parichha*[1], Varun Suresh*[1], Mallika Chatterjee[1$], Aditya Kshirsagar[2], Lihi Ben-Reuven[2], Tsviya Olender[2], M. Mark Taketo[3], Velena Radosevic[4,5], Mihaela Bobic-Rasonja[4,5], Sara Trnski[4], Michael J. Holtzman[6], Nataša Jovanov Milošević[#4,5], Orly Reiner[#2], Shubha Tole[#1]

[1]Department of Biological Sciences, Tata Institute of Fundamental Research, Mumbai 400005, India; [2]Department of Molecular Genetics, Weizmann Institute of Science, Israel; [3]Division of Experimental Therapeutics Graduate School of Medicine, Kyoto University (Yoshida-Konoé-Cho, Sakyo), Kyoto 606-8501, Japan; [4]Croatian Institute for Brain Research, Department of Medical Biology, School of Medicine University of Zagreb, Šalata 12, Zagreb, Croatia, [5]University Hospital Centre Zagreb, Department of Gynecology and Department of Pathology and Cytology, Petrova 13, Zagreb, Croatia; [6]Pulmonary and Critical Care Medicine, Washington University, St. Louis, Missouri, USA 63110.

*These authors contributed equally to this work

[#]Authors for correspondence: stole@tifr.res.in; orly.reiner@weizmann.ac.il; njovanov@gmail.com

[$]Current address: Amity Institute of Neuropsychology and Neurosciences, Amity University, Noida, India.

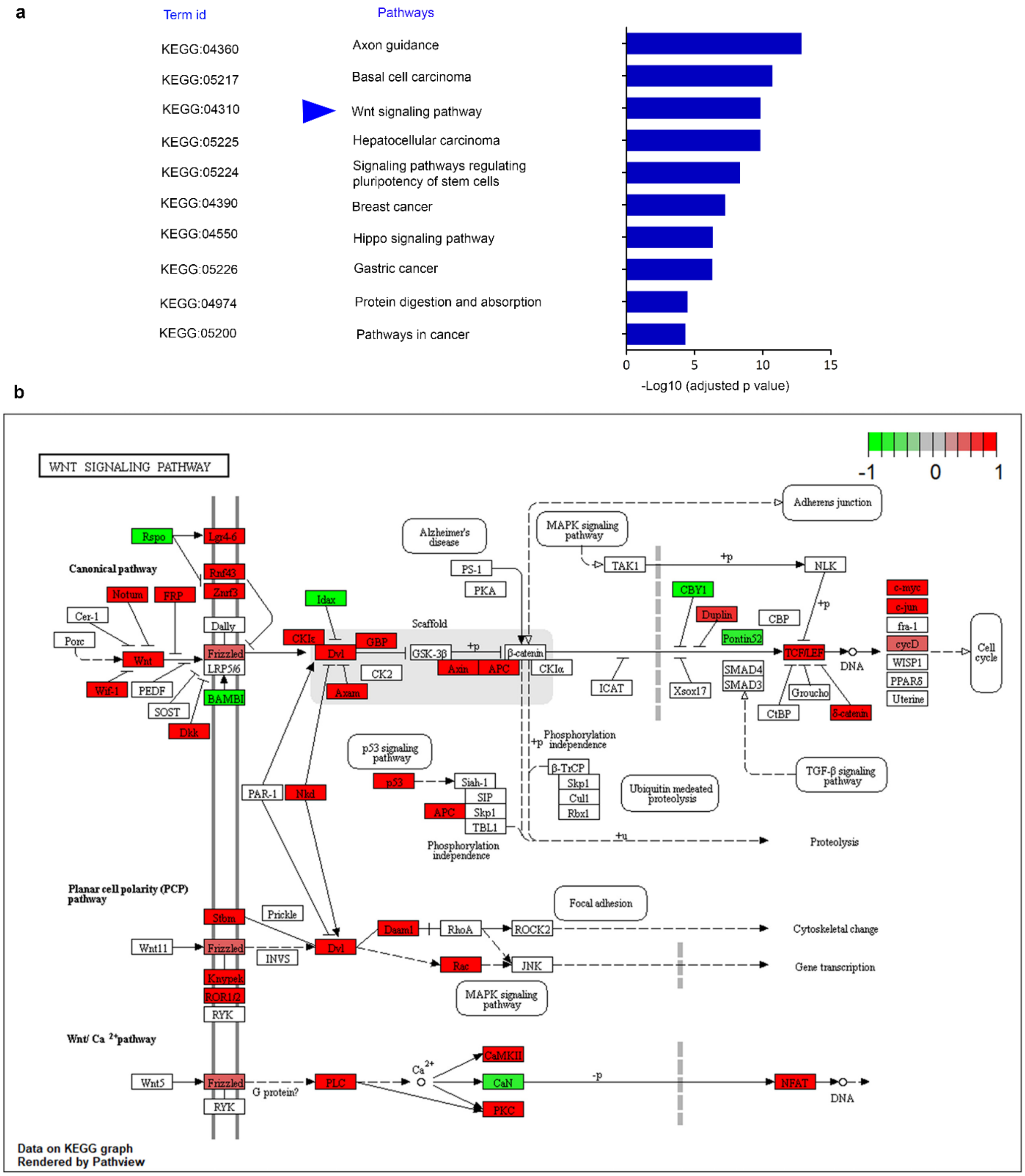

Supplementary Figure S1: Pathway analysis from differentially expressed gene sets in the control and Lmx1aCre::β-catenin GOF ChP shows upregulation of Wnt responsive genes. (A) KEGG pathway analysis identified the Wnt signaling pathway (blue arrowhead) among the top 10 dysregulated pathways. (B) Analysis of differentially expressed genes in the canonical Wnt signaling pathway and the planar cell polarity pathway in the control and β-CATENIN GOF ChP. Source data are provided as a Source Data file.

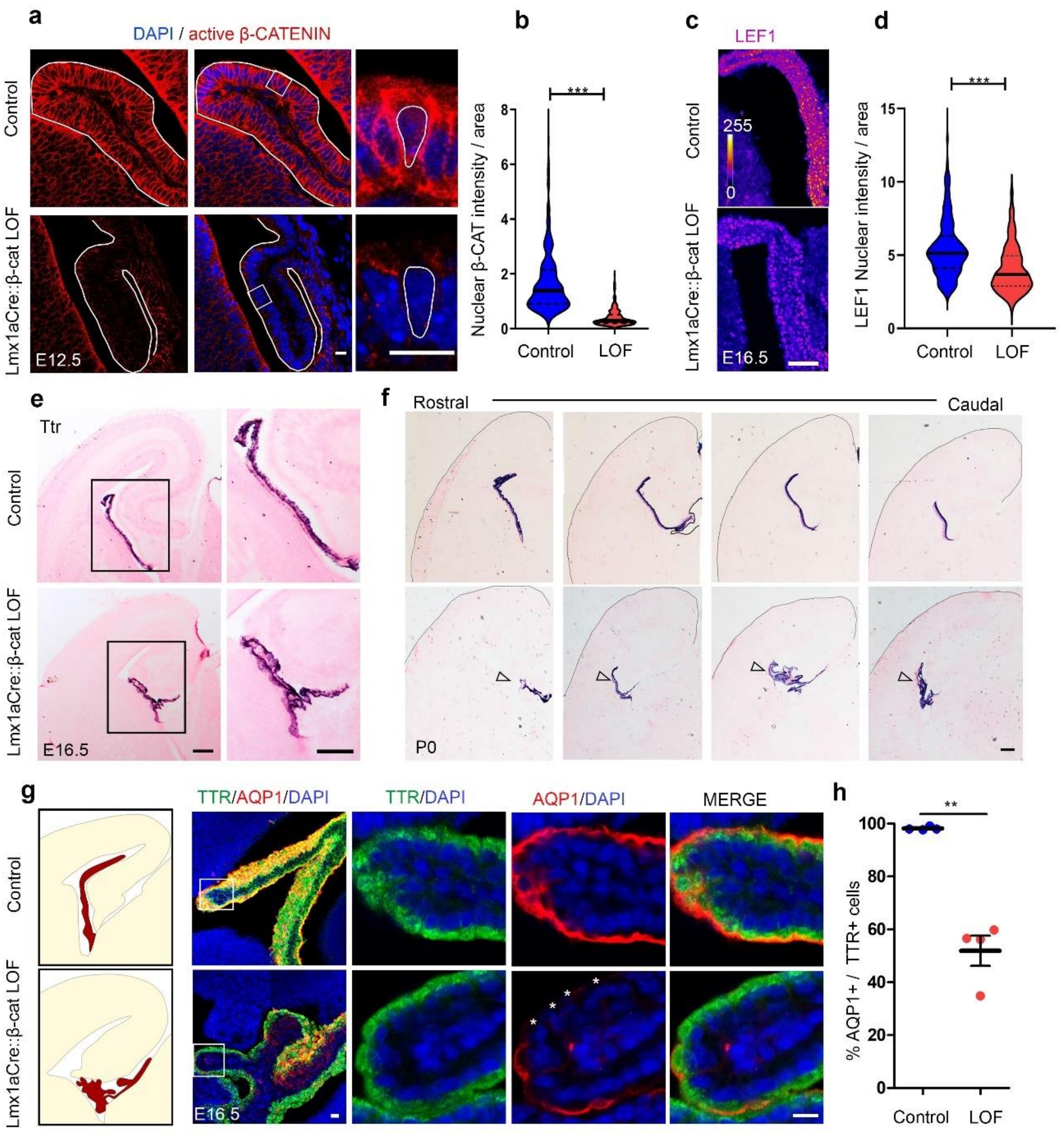

Supplementary Figure S2. Loss of canonical Wnt signaling (β-CATENIN LOF) in the hem and ChPe disrupts choroid plexus development. (a) At E12.5, nuclear localization of active (non-phosphorylated) β-CATENIN was significantly reduced in the Lmx1aCre::β-cat LOF ChPe compared with controls as seen by (a) immunohistochemistry and (b) a violin plot representing quantitation of n=329 nuclei (control) and n=302 nuclei (LOF); N=3 brains (biologically independent replicates) for each genotype examined over 2 independent experiments. (c, d) At E16.5, LEF1 levels decrease (c), shown in a violin plot (d) representing quantitation of n=401 nuclei (control) and 400 nuclei (LOF); N=3 brains (biologically independent replicates) for each genotype examined over 3 separate experiments. (e, f) *Ttr* mRNA expression is maintained upon loss of β-CATENIN signaling at E16.5 and P0, even though there is substantial dysmorphia, N=3 brains (biologically independent replicates) for each genotype examined over 2 independent experiments. (g, h) At E16.5, only 51.9 % of the TTR+ cells also display AQP1 (white asterisk), N=4 brains (biologically independent replicates) for each genotype examined over 4 independent experiments. Boxed regions (a, e, g) are shown at high magnification in the adjacent panels.

(b, d, and h) Statistical test: two-tailed unpaired Student's t-test with unequal variance; for violin plot (b, d), p<0.0001, for scatter plot (h), p=0.004, * p < 0.05, ** p < 0.01, *** p < 0.001, ns if p-value > 0.05. For violin plots (b,d) solid black line represents median and dotted lines represents quartiles, for scatter plot (h) bars represent mean±SEM. Color key in (c): dark blue (grey value=0) and white (grey value=255). Scale bars: 10 μm (all panels in a, g); 50 μm (all panels in c); 100 μm (all panels in e, f). Further information on replicates and reproducibility for this figure is mentioned in the "Statistics and Reproducibility" section of the Methods. Source data are provided as a Source Data file.

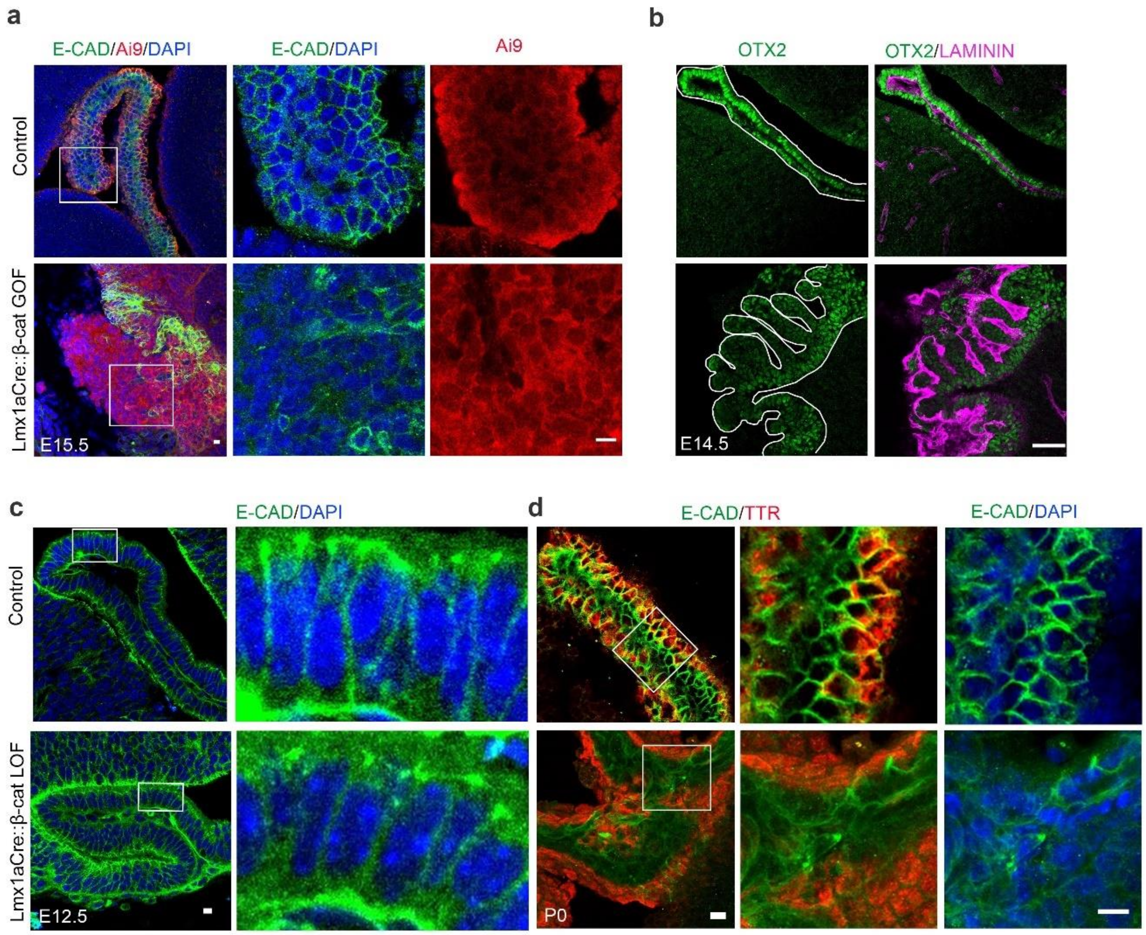

Supplementary Figure S3: Cell adhesion changes upon gain or loss of β-CATENIN.
(a, b) At E14.5-E15.5, adherens junction marker E-CADHERIN (a) appears to be disorganized in the Lmx1aCre::β-cat GOF ChP compared with the control. Co-immunostaining for LAMININ and OTX2 (b) reveals a highly dysmorphic ChP with multiple folds and apparently reduced OTX2 in Lmx1aCre::β-cat GOF embryos. (c) E-CADHERIN distribution appears similar at E12.5 in the Lmx1aCre::β-cat LOF ChP and control ChP. By birth (d) E-CADHERIN is disorganized in the β-cat LOF ChP which appears dysmorphic, although TTR labeling is maintained. (a-d) Representative images of sections taken from N=3 brains (biologically independent replicates) for each genotype examined over 3 independent experiments. Boxed regions (a, c, d) are shown at high magnification in the adjacent panels. Scale bars: 10 μm (all panels in a, c and d); 50 μm (all panels in b).

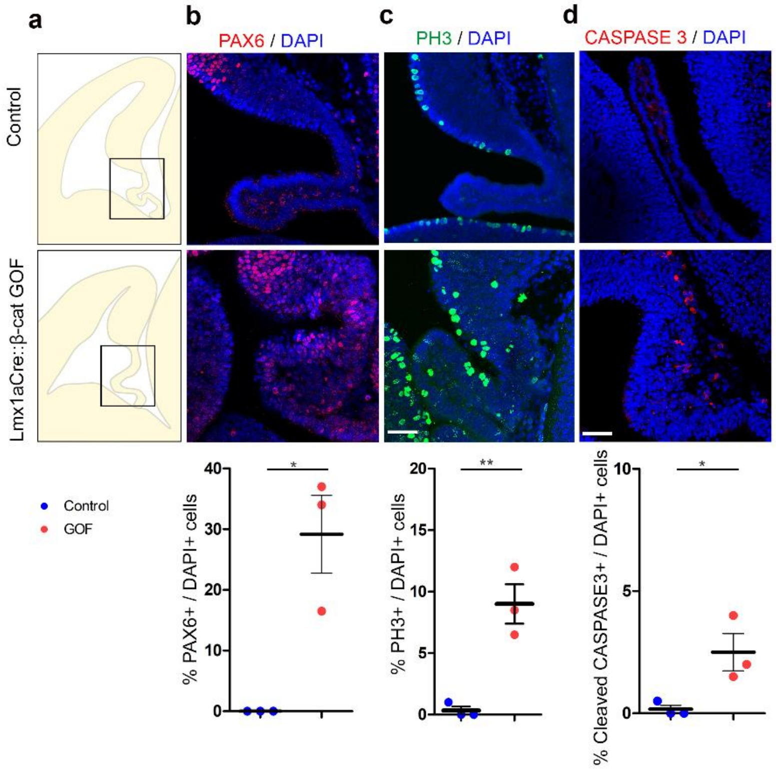

Supplementary Figure S4: Proliferation and apoptosis in the choroid plexus upon stabilization of β-CATENIN. (a) Cartoons of E12.5/E13.5 mouse brain coronal sections showing the choroid plexus (Boxed region). (b) The neuronal apical progenitor marker PAX6 is present in 30% of the DAPI+ nuclei in the Lmx1aCre::β-cat GOF ChP, whereas the control ChP does not display PAX6 labeling. (c) The cell proliferation marker PH3 labels 9% of the 422 DAPI+ cells scored in Lmx1aCre::β-cat GOF ChP; while only 0.3% of the 400 DAPI+ cells are labeled in the control. (d) The apoptosis marker cleaved CASPASE 3 is detected in 2.5% of the 600 DAPI+ cells in Lmx1aCre::β-cat GOF ChP and in 0.2% of 576 DAPI+ cells in the control. For (b-d) N=3 brains (biologically independent replicates) for each genotype examined over 2 separate experiments. Boxed regions (a) are shown at high magnification in the adjacent panels. Bars in scatter plots (b, c, and d) represent mean±SEM. Statistical test: two-tailed unpaired Student's t-test with unequal variance; $p=0.0103$ (b, PAX6), $p=0.0064$ (c, PH3), $p=0.0405$ (d, CASPASE3); * $p < 0.05$, ** $p < 0.01$, *** $p < 0.001$, ns if p-value > 0.05. Scale bars: 100 µm (all panels in C and D).

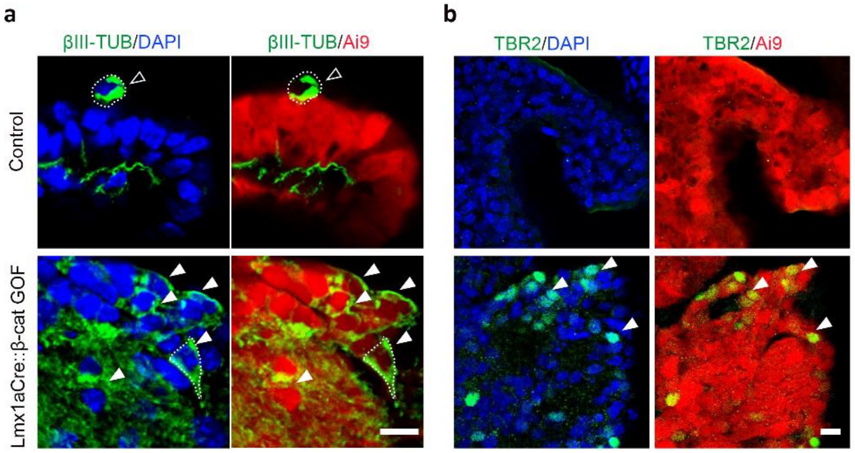

Supplementary Figure S5: Neurons appear in the choroid plexus upon stabilization of β-CATENIN derived from the Lmx1a lineage. (a) At E16.5 a few βIII TUBULIN positive neurons are present in the control (Lmx1aCre::Ai9) ChP, but these do not co-localize with Ai9 and therefore do not stem from the Lmx1a lineage (open arrowheads, a). In contrast, multiple βIII TUBULIN-positive neurons and TBR2-positive cells are also Ai9-positive in Lmx1aCre::β-cat GOF::Ai9 ChPe (arrowheads, a,b). (a,b) Representative images of sections taken from N=5 brains (biologically independent replicates) for each genotype examined over 5 independent experiments. Scale bars: 10 μm (all panels in a and b).

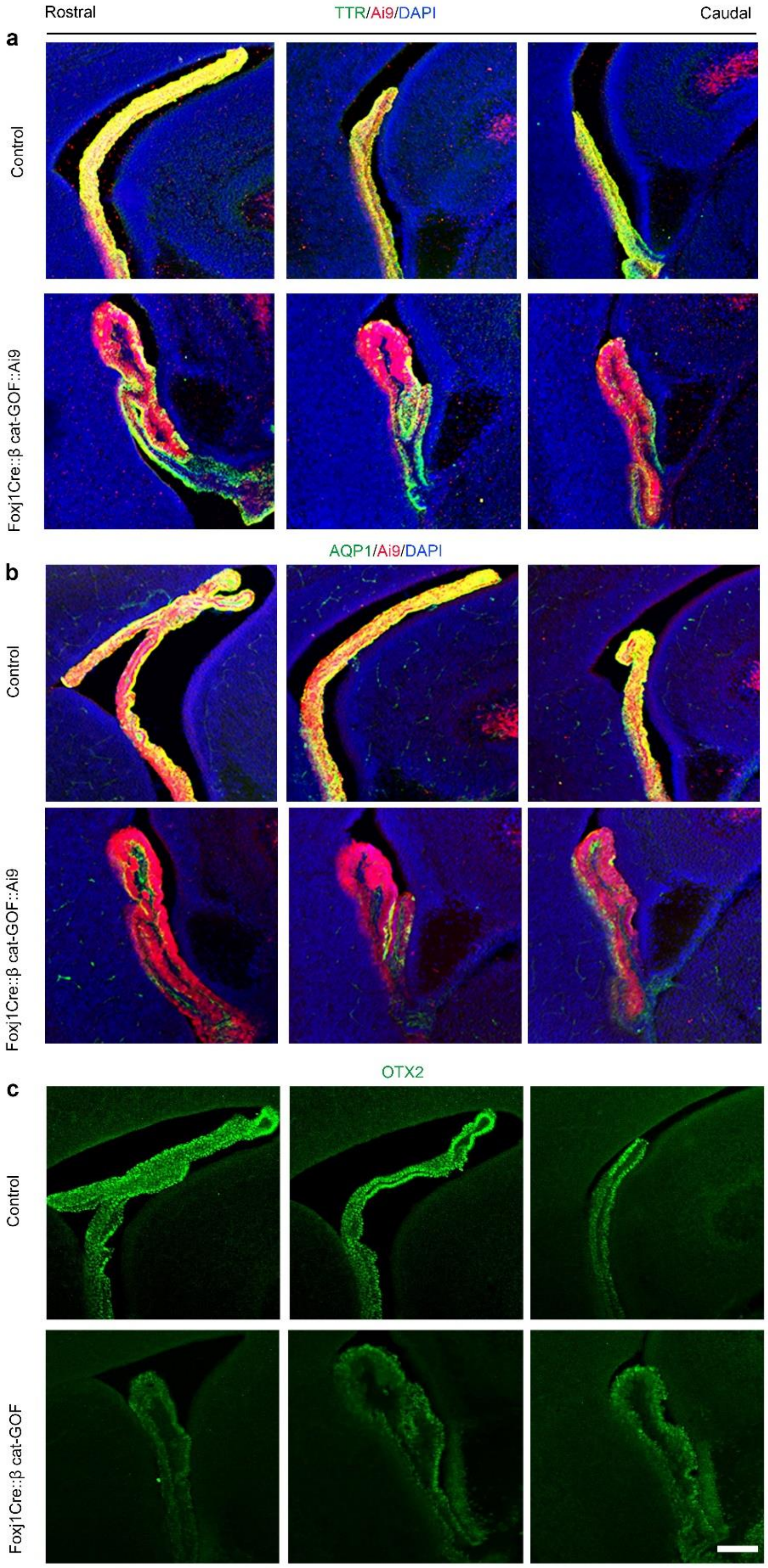

Rostral
TTR/Ai9/DAPI
Caudal
a
Control
Foxj1Cre::β cat-GOF::Ai9
AQP1/Ai9/DAPI
b
Control
Foxj1Cre::β cat-GOF::Ai9
OTX2
c
Control
Foxj1Cre::β cat-GOF

Supplementary Figure S6: Stabilizing β-CATENIN selectively in the specified ChPe using FoxJ1Cre leads to loss of ChPe fate. Rostrocaudal sections from E16.5 control and Foxj1Cre::β-cat GOF embryos reveal TTR (a) and AQP1 (b) is lost from some regions of the ChP, whereas OTX2 (c) persists at lower levels in Foxj1Cre::β-cat GOF brains. (a, b and c) Representative images of sections taken from N=5 brains (biologically independent replicates) for each genotype examined over 4 independent experiments. Scale bar: 100 μm (all panels in a, b and c).

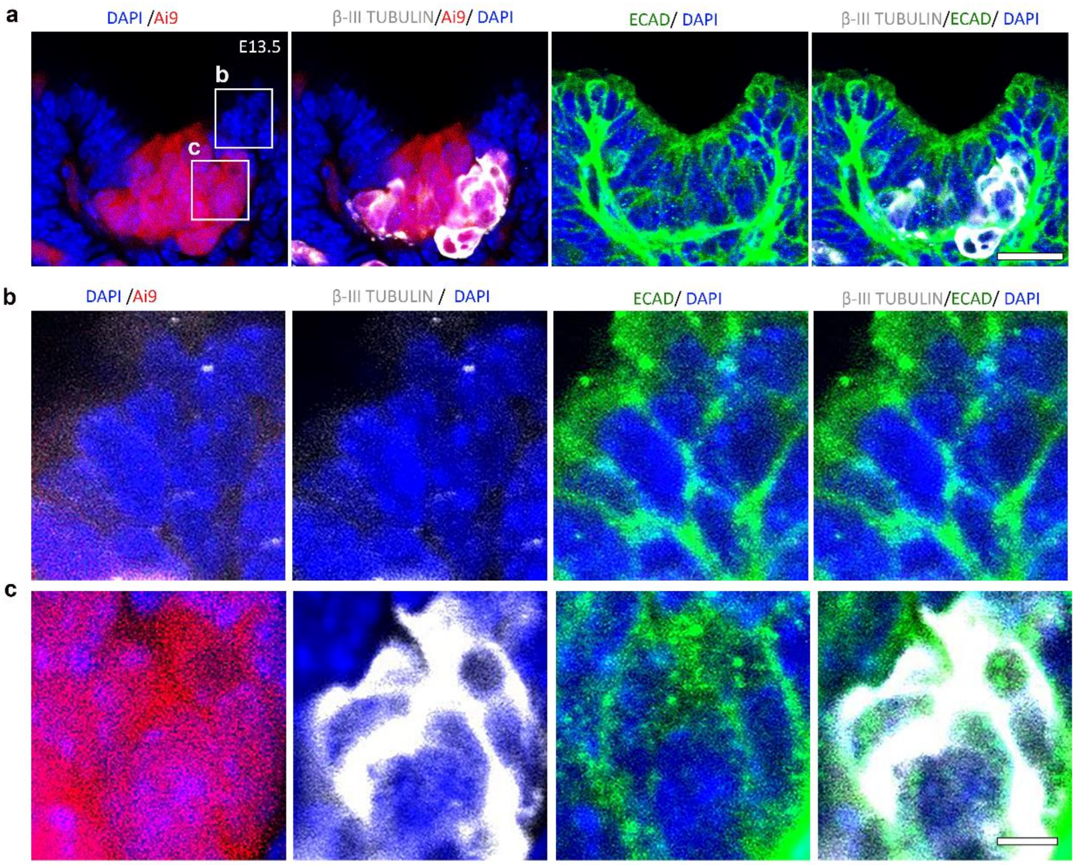

Supplementary Figure S7: β-Catenin GOF causes upregulation of β-III TUBULIN in E-CADHERIN-positive ChPe cells. (a) Lmx1aCre::β-Catenin GOF::Ai9 female ChPe display mosaic Ai9+ patches interspersed with Ai9-patches. βIII TUBULIN appears selectively in the Ai9+ patches whereas E-CADHERIN staining is seen in both Ai9+ and Ai9- cells. Boxed regions (a) are shown at high magnification in (b) and (c). (b) Ai9- internal control cells, do not display detectable βIII TUBULIN but contain E-CADHERIN. (c) Ai9+ cells display co-localization of β-III TUBULIN and E-CADHERIN. (a-c) Representative images of sections taken from N=3 brains (biologically independent replicates) for each genotype examined over 2 independent experiments. Scale bars: 5 μm (all panels in b and c); 20 μm (all panels in a).

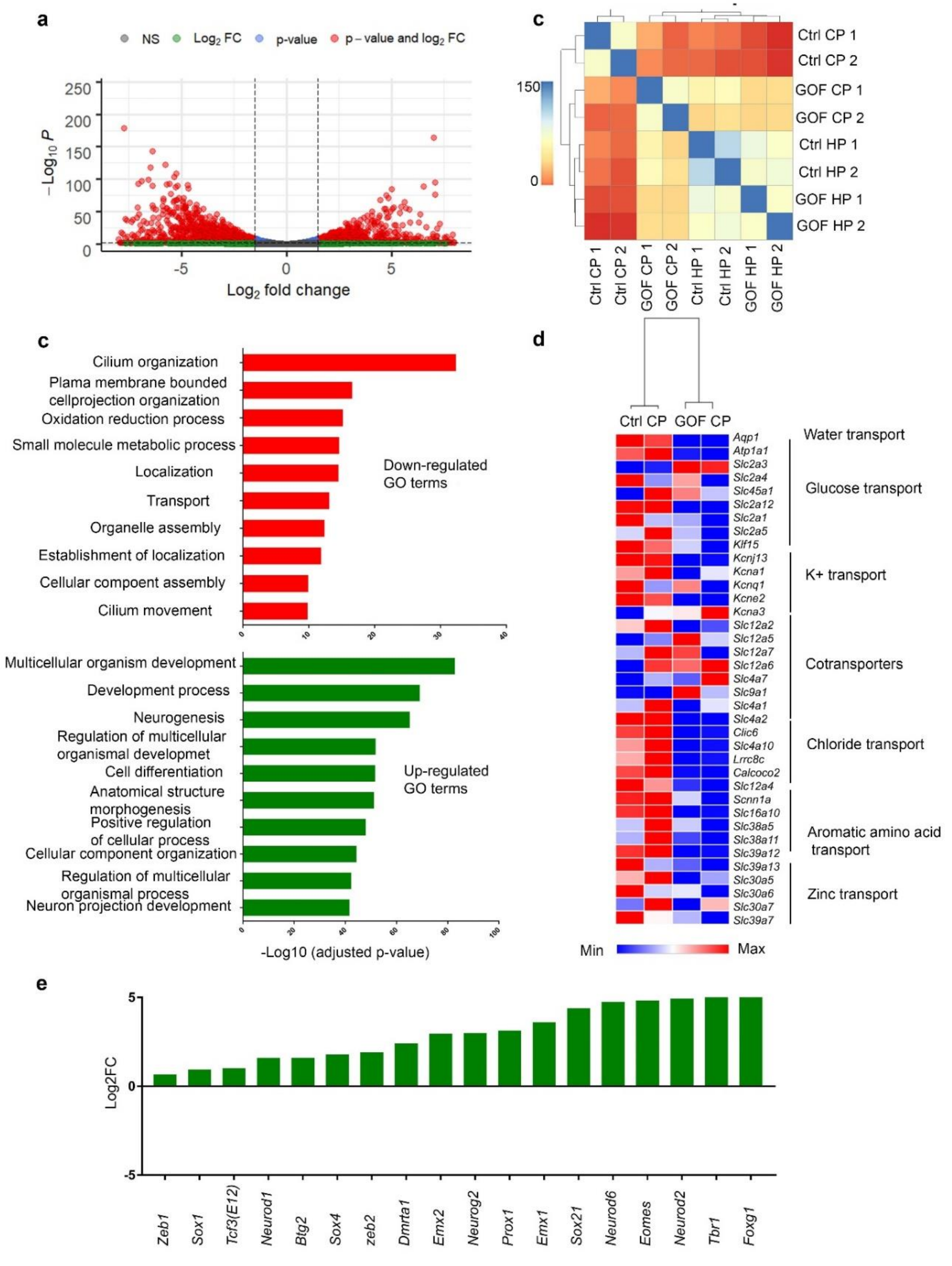

Supplementary Figure S8: Analysis of differentially expressed gene sets in the control and Lmx1aCre::β-catenin GOF ChP and Hippocampus. (a) Volcano plot and (b) GO analysis of differentially expressed genes in the control and β-catenin GOF ChP (c) Sample correlation matrix shows that the β-catenin GOF ChP is more similar to the control hippocampus (HP) than it is to the control ChP. Color code: Blue (high correlation), orange (low correlation). (d) Heatmap of normalized read counts of a curated set of genes encoding different classes of transporters expressed in the normal ChP reveals they

are uniformly downregulated in the β-catenin GOF ChP compared with the control. (e) Several pro-neuronal transcription factors belonging to the bHLH, Sox, and Zeb families are upregulated in Lmx1aCre::β-catenin GOF ChP. Color bars in (d): blue (low expression), red (high expression). Source data are provided as a Source Data file. For RNAseq experiments (a-e) N=2 biologically independent replicates.

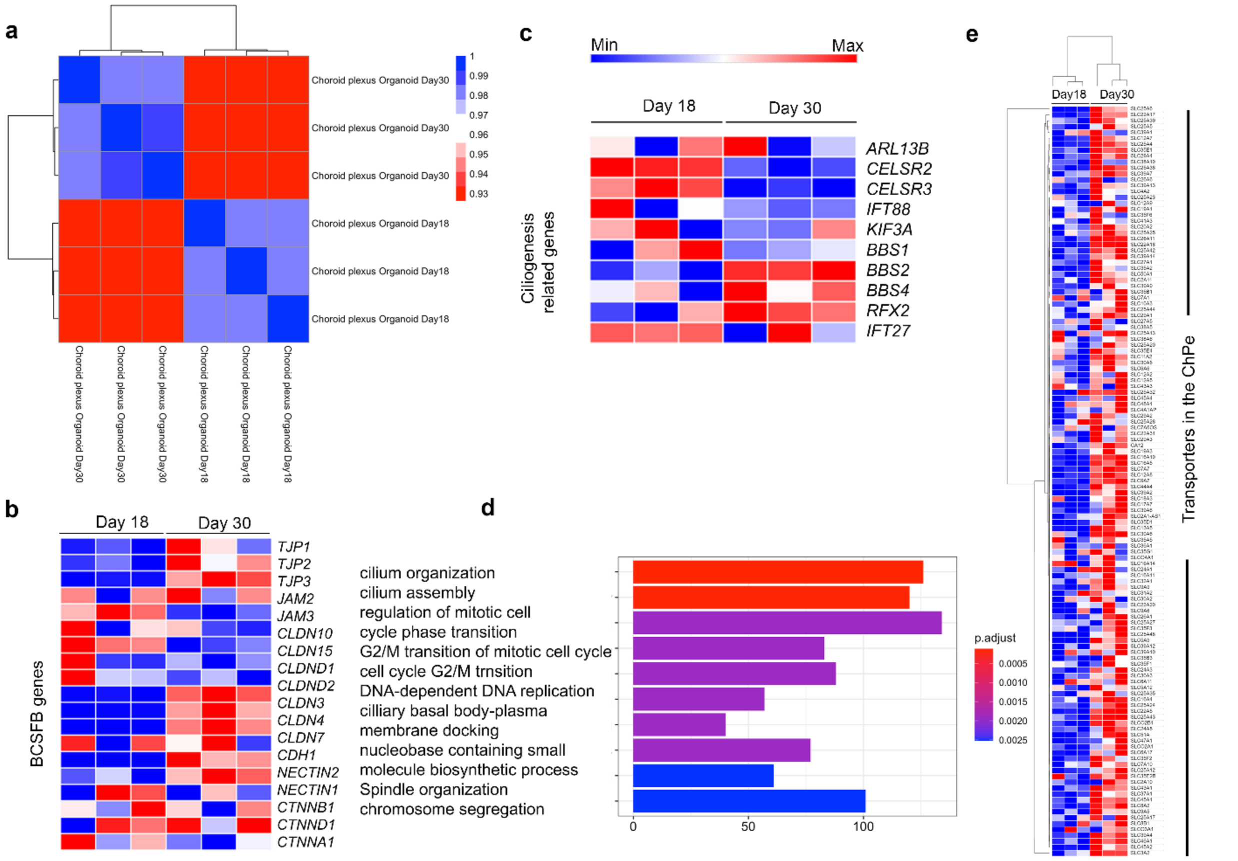

Supplementary Figure S9: Characterization of ChPe-like differentiation in hESC organoids. (a-e) A comparison of transcriptomics data from day 18 and day 30 organoids; N=3 biologically independent replicates. (a) Sample correlation matrix, color key: high correlation (blue) and low correlation (red) (b, c). Heat map of the normalized read counts reveals that several BCSF genes are upregulated (b), and ciliogenesis-related genes are differentially regulated (c). (d) GO analysis (e) Heat map plotted from normalized read counts shows an overall upregulation of transporter genes (belonging to the Slc gene family) in day 30 organoids. Color bars in (b, c,e): blue (low expression), red (high expression). Source data are provided as a Source Data file.

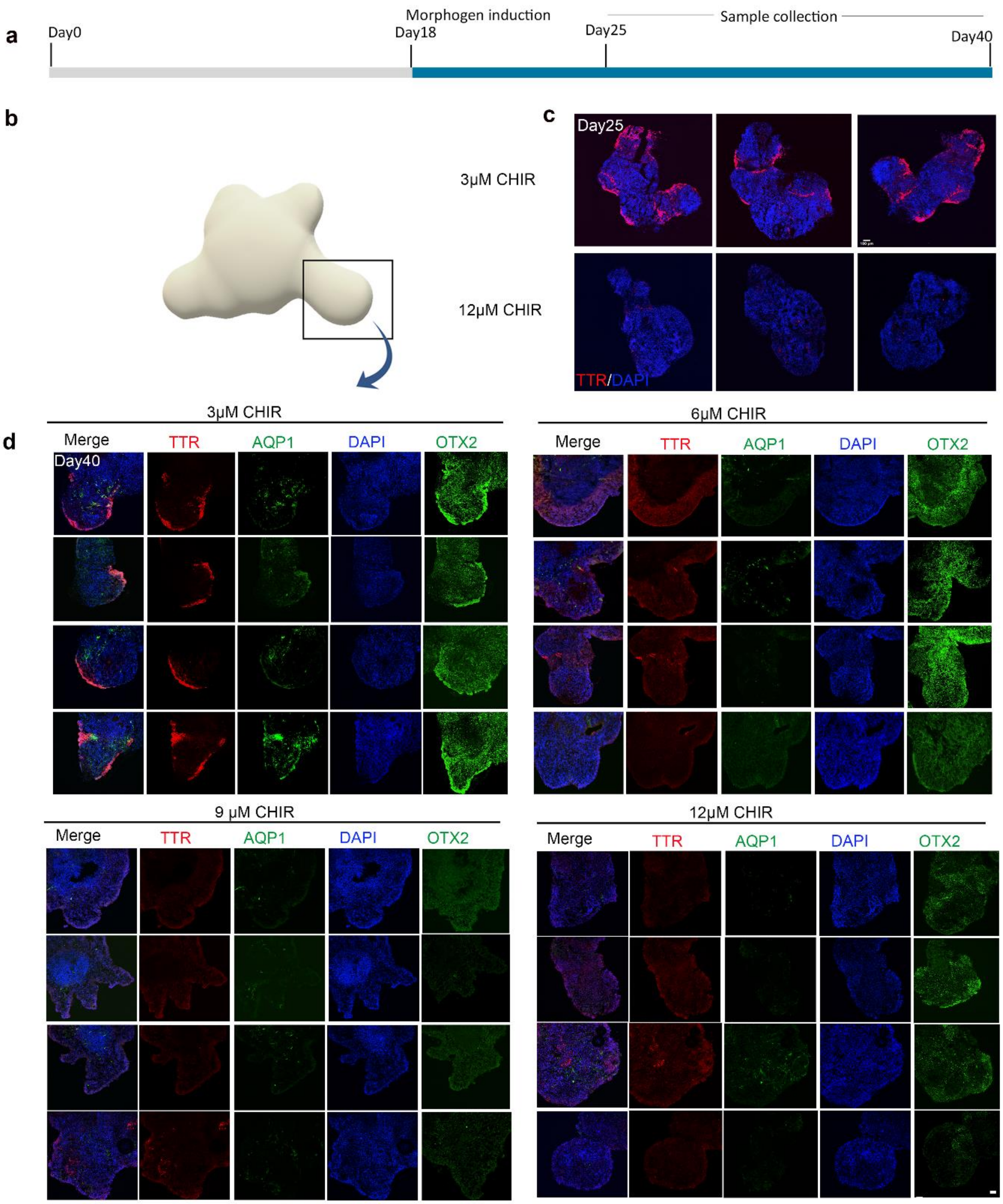

Supplementary Figure S10: High activation of the canonical Wnt pathway in hESC-derived organoids causes suppression of ChPe markers. Additional examples of data shown in Figure 10 c and d. (a) Scheme showing the protocol for treatment with canonical Wnt agonist CHIR (c) ChP marker TTR is present in the periphery of day 25 organoids exposed to 3 μM, but not 12 μM CHIR, from day 18 N=3 organoids (biologically independent replicates) examined over 2 independent experiments. (d) High magnification images (boxed region shown in b) of serial sections of four examples of day 40 organoids exposed to 3 μM reveal TTR, AQP1, and OTX2 labeling in the periphery. This staining progressively

decreases and is undetectable upon increasing exposure to higher concentrations of CHIR (6/ 9/ 12 μM). For (d) N=14 organoids (3 μM CHIR), 9 organoids (6 μM CHIR), 5 organoids (9 μM CHIR treated), and 14 organoids (12 μM CHIR treated) from 2 independent experiments were used for analysis (each organoid was considered as an independent biological replicate). Scale bar: 100 μm (all panels in c and d).

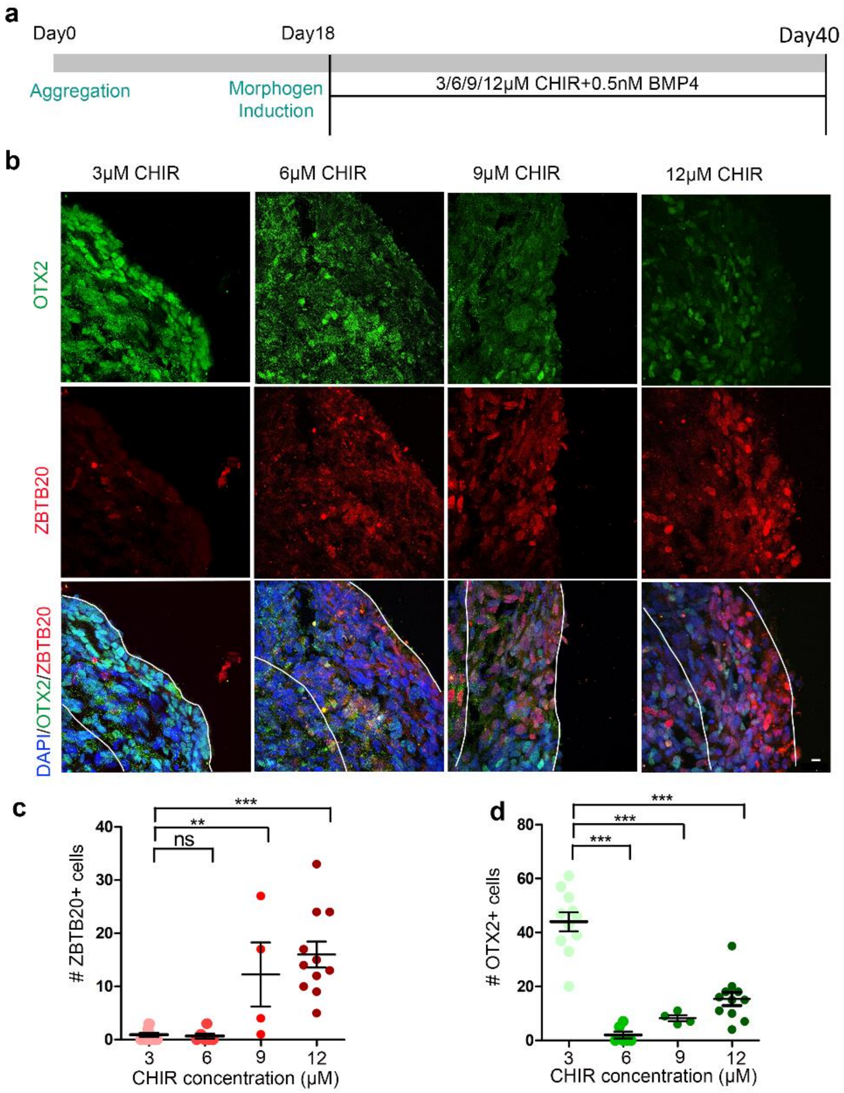

Supplementary Figure S11: Hippocampus-specific marker ZBTB20 is upregulated, and OTX2 is downregulated upon high activation of the canonical Wnt pathway in hESC-derived organoids. (A) Schematic showing the organoid culture protocol and experimental design. (B) Immunofluorescence images of the periphery of an organoid display decreasing OTX2 and increasing ZBTB20 labeling upon exposure to increasing concentrations of CHIR. (C, D) Quantification of the number of ZBTB20+ and OTX2+ cells in organoids treated with 6/ 9/12 μM CHIR. N=11organoids (3 and 12 μM CHIR); 6 organoids (6 μM CHIR); 4 organoids (9 μM CHIR) examined over 2 independent. Bars in scatter plots (c,d) represent mean±SEM. Statistical tests in c and d: two-tailed unpaired Student's t-test with unequal variance. For (c), p= 0.7004 (3 μM vs 6 μM CHIR), p= 0.0057 (3 μM vs 9 μM CHIR), p < 0.0001 (3 μM vs 12 μM CHIR), and 14 organoids (12 μM CHIR), (d), p < 0.0001 (3 μM vs 6/9/12 μM CHIR). * p < 0.05, ** p < 0.01, *** p < 0.001, ns if p-value > 0.05. Scale bar: 10 μm (all panels in b).
