# Supplementary material for: Constitutive activation of canonical Wnt signaling disrupts choroid plexus epithelial fate
Source: Nat Commun. 2022 Feb 2;13:633. doi: 10.1038/s41467-021-27602-z (PMC8810795; doi:10.1038/s41467-021-27602-z)
Supplement: Supplementary file 2 — Description of Additional Supplementary Files [file 41467_2021_27602_MOESM2_ESM.docx]

Description of Supplementary data 1

Title: Supplementary Data 1.

Description: RNASeq data from mouse and organoid. (Tab1) RNASeq data (base mean, Log2 Fold change, LfcSE, padj) from control and Lmx1aCre::β-Catenin GOF Choroid plexus (E14.5), (Tab2) RNASeq data (base mean, Log2 Fold change, LfcSE, padj) from Control and Lmx1aCre::β-Catenin GOF Hippocampus (e14.5), (Tab3) Normalized read files from top 500 differentially regulated genes from control and Lmx1aCre::β-Catenin GOF choroid plexus compared with control Hippocampus. (Tab4) RNASeq data (base mean, Log2 Fold change, LfcSE, padj) from Day 18 vs Day 30 ChP organoid. (Tab5) RNASeq data (base mean, Log2 Fold change, LfcSE, padj) from 3µm vs 12µm CHIR treated Day 30 ChP organoids. (Tab6) Comparison of Control and Lmx1aCre::β-Catenin GOF Choroid plexus RNAseq data with Dani et al., 2021.

.
