# Supplementary material for: Constitutive activation of canonical Wnt signaling disrupts choroid plexus epithelial fate
Source: Nat Commun. 2022 Feb 2;13:633. doi: 10.1038/s41467-021-27602-z (PMC8810795; doi:10.1038/s41467-021-27602-z)
Supplement: Supplementary file 4 — Reporting Summary [file 41467_2021_27602_MOESM4_ESM.pdf]

nature research

Corresponding author(s): Shubha Tole, Orly Reiner, and Nataša Jovanov Miloševic.

Last updated by author(s): 15/11/2021

Reporting Summary

Nature Research wishes to improve the reproducibility of the work that we publish. This form provides structure for consistency and transparency in reporting. For further information on Nature Research policies, see our Editorial Policies and the Editorial Policy Checklist.

Statistics

For all statistical analyses, confirm that the following items are present in the figure legend, table legend, main text, or Methods section.

n/a	Confirmed	
☐	☒	The exact sample size (*n*) for each experimental group/condition, given as a discrete number and unit of measurement
☐	☒	A statement on whether measurements were taken from distinct samples or whether the same sample was measured repeatedly
☐	☒	The statistical test(s) used AND whether they are one- or two-sided<br>*Only common tests should be described solely by name; describe more complex techniques in the Methods section.*
☒	☐	A description of all covariates tested
☐	☒	A description of any assumptions or corrections, such as tests of normality and adjustment for multiple comparisons
☐	☒	A full description of the statistical parameters including central tendency (e.g. means) or other basic estimates (e.g. regression coefficient) AND variation (e.g. standard deviation) or associated estimates of uncertainty (e.g. confidence intervals)
☐	☒	For null hypothesis testing, the test statistic (e.g. *F*, *t*, *r*) with confidence intervals, effect sizes, degrees of freedom and *P* value noted<br>*Give P values as exact values whenever suitable.*
☒	☐	For Bayesian analysis, information on the choice of priors and Markov chain Monte Carlo settings
☒	☐	For hierarchical and complex designs, identification of the appropriate level for tests and full reporting of outcomes
☒	☐	Estimates of effect sizes (e.g. Cohen's *d*, Pearson's *r*), indicating how they were calculated

Our web collection on statistics for biologists contains articles on many of the points above.

Software and code

Policy information about availability of computer code

Data collection	Illumina Hiseq Platform was used for Next generation sequencing. Imaging data collection was performed on Zeiss Axioskop-2 plus microscope equipped with a Nikon DS-fi2 camera and associated software (Nis Elements 4.0), mouse sections were imaged in Olympus FluoView 1200 confocal microscope and the human brain sections were imaged in Olympus FluoView 3000 confocal microscope. All the organoid sections were imaged using the Andor Dragonfly spinning disk confocal microscope system. All the image analysis was done on Fiji-ImageJ, Imaris (Version 7.2.3) and/or Adobe Photoshop CS6. All statistical analysis were performed in Graph pad prism (V 7.0 & V 9.1.0). qPCR was performed in LightCycler® 96 and QuantStudio 5 Real-time PCR system.
Data analysis	Mouse RNA-Seq analysis was sequenced on the Illumina platform to achieve 100bp or 150bp reads to generate 30 Million paired-end reads per sample. FastQ QC was performed as described in (Andrews, S 2010), and reads > 30 Phred scores were aligned using HISAT2 (D.Kim et al., 2019). Feature counts were used to quantify the number of reads per transcript. Differential expression analysis was performed using DESeq2 (Love et al., 2014) on the R platform (v3.6.3). Genes showing |log2 Fold change|≥1 were used for further analysis. Gene ontology analysis was performed using geneontology.org or gprofiler.org. Semantics were summarized using "REVIGO" (Supek et al., 2011), and bar plots were created with GraphPad Prism V7.0 for Windows, GraphPad Software, San Diego California USA, www.graphpad.com). Heat maps and sample correlation plots were plotted using R studio. Gene-based heatmaps were plotted using normalized reads on Morpheus (Morpheus, https://software.broadinstitute.org/morpheus)<br>For Organoid RNA-Seq, Organoids generated in figure 6 and supplementary figure 2, were pooled such that for one biological replicate 3 organoids were used and three such biological replicates were performed for day 18 and day 30 organoids. Bulk MARS-seq libraries were produced from 50ng of total RNA as previously described (Meador et al.,2014). Libraries were then sequenced with 75bp single-end read on Illumina Nextseq500 platform and FASTQ files were processed and analyzed using UTAP (Kohen et al., 2019). Briefly, sequenced reads were trimmed using cutadapt (parameters: -a adaptor -a "A {10}" –times 2 -u 3 -u -3 -q 20 -m 25) (M Martin., 2011) and were mapped to hg38 indexed reference genome using STAR (Dobin et al., 2013) v2.4.2a with the following parameters: –alignEndsType EndToEnd, –out Filter Mismatch Nover Lmax 0.05, –two pass Mode Basic). UMIs were counted after marking duplicates using HTSeq-count (Anders et al., 2015) in union mode. For the number of reads per gene, 1000bp of 3' end Gencode annotated transcripts were counted. The total RNA-seq from 3 µM

and 12 μM CHIR treated organoids (Figure 7) were done using TruSeq stranded mRNA library kit and sequenced by DNA Link Sequencing Lab, Korea, on NovaSeq 6000, 100PE. Data were analyzed with the UTAP pipeline (Kohen et al., 2019), where adapter trimming and reference genome alignment was performed as described above. Read count per gene was done with STAR. Normalization of the counts and differential expression analysis was performed using DESeq2 V3.14 (Love et al., 2014) with the parameters: betaPrior=True, cooks Cutoff=FALSE, independentFiltering=FALSE. Raw P values were adjusted for multiple testing using the procedure of Benjamini and Hochberg. Genes with log2FC >1 or <-1, p adjust < 0.05 and baseMean > 10 were considered as differentially expressed. GO Biological process overrepresentation test on differentially expressed genes between different time points of organoid growth was performed with the package clusterProfiler (Yu et al., 2012).

For manuscripts utilizing custom algorithms or software that are central to the research but not yet described in published literature, software must be made available to editors and reviewers. We strongly encourage code deposition in a community repository (e.g. GitHub). See the Nature Research guidelines for submitting code & software for further information.

Data

Policy information about availability of data

All manuscripts must include a data availability statement. This statement should provide the following information, where applicable:

- Accession codes, unique identifiers, or web links for publicly available datasets
- A list of figures that have associated raw data
- A description of any restrictions on data availability

The mouse RNA-seq data (Figure 7,8, Supplementary figure s1& 8) generated in this study have been deposited in the GEO database under accession code: GSE162784 (https://www.ncbi.nlm.nih.gov/geo/query/acc.cgi?acc=GSE162784) and the organoid MARS-seq and RNA-seq datasets (Figure 9,10, Supplementary figure s9) have been deposited in the GEO database under accession code GSE162808 (https://www.ncbi.nlm.nih.gov/geo/query/acc.cgi?acc=GSE162808) . Raw data for Figure 1e & j, 2c, e, g & i, 3b, e, g,h & j, 4c, e & h, 5d & e, 6 i, k & m, 10 e & f , Supplementary figure s2b, d &h, s4 b, c & d, s11 c & d are available in the source data file.

Field-specific reporting

Please select the one below that is the best fit for your research. If you are not sure, read the appropriate sections before making your selection.

☒ Life sciences ☐ Behavioural & social sciences ☐ Ecological, evolutionary & environmental sciences

For a reference copy of the document with all sections, see nature.com/documents/nr-reporting-summary-flat.pdf

Life sciences study design

All studies must disclose on these points even when the disclosure is negative.

Sample size	Sample-size calculation was not performed. Sample sizes were chosen based on published studies (Langford et al., 2020, Kaiser et al., 2021) that used the same techniques and are reported in figure legends.
Data exclusions	For Lmx1aCre animals, since the transgene is on the X-Chromosome, females showed mosaic expression of the Cre recombinase due to random X inactivation. Cre-positive male embryos/pups were used for analysis and females were excluded, except for the experiments in Figure 6 and Supplementary Figure S7 for which female embryos were analyzed.
Replication	All mouse experiments were conducted using embryos/mice from least two independent litters and performed on different days. Results from all replicates are consistent and represented in the study. For the hESC organoid studies, 3 independent batches of organods (experiments performed in different days) were used and all replicates gave consistent results.
Randomization	Randomization is not necessary or applicable to our study our approaches as all of our data were well-controlled wild type vs mutant/ treatment comparisons. Embryos were allocated into age matched groups based on genotypes. Where possible, littermate controls were used.
Blinding	The authors were not blinded to the genotype of samples. The genotypes could be distinguished by apparent phenotypic features, so it was not possible to perform the quantifications in a blinded manner.

Reporting for specific materials, systems and methods

We require information from authors about some types of materials, experimental systems and methods used in many studies. Here, indicate whether each material, system or method listed is relevant to your study. If you are not sure if a list item applies to your research, read the appropriate section before selecting a response.

Materials & experimental systems

n/a	Involved in the study
	x Antibodies
	x Eukaryotic cell lines
x	Palaeontology and archaeology
	x Animals and other organisms
	x Human research participants
x	Clinical data
x	Dual use research of concern

Methods

n/a	Involved in the study
x	ChIP-seq
x	Flow cytometry
x	MRI-based neuroimaging

Antibodies

Antibodies used

Primary antibodies :
(1) LEF1 (Rabbit, CST catalogue # C12A5, Lot # 8)
(2) β-CATENIN (Mouse, BD biosciences catalogue #610153, Lot # 7187864)
(3) RFP (Rabbit, Abcam catalogue # ab62341, Lot # GR33231879-5)
(4) FZD1 (Rabbit, catalogue # LS-A4150, Lot # 5502)
(5) AXIN2 (Rabbit, Abcam catalogue #ab185821, Lot # GR32276513)
(6) TTR (Rabbit, DAKO catalogue # A0002, Lot # 20049834)
(7) AQP1 (Mouse SCBT catalogue # sc25287, Lot # L0619)
(8) OTX2 (Mouse, SCBT catalogue # sc-514195)
(9) OTX2 (Invitrogen, catalogue # PA5-39887, Lot # TI2625635A)
(10) AQP1 (Rabbit, Invitrogen catalogue # MA5-32593, Lot # TI2638171)
(11) β-III TUBULIN (Rabbit, CST catalogue # 5666, Lot 2)
(12) β-CATENIN (Mouse monoclonal, catalogue # M3539, Dako Agilent, Lot # 3)
(13) Non-phospho (Active) β-CATENIN (Rabbit, CST catalogue # D13A1)
(14) OTX2 (Mouse, catalogue # MA5-15854)
(15) SOX9 (Rabbit, Abcam catalogue # ab1852, Lot # GR3300369)
(16) AQP1 (Rabbit, invitrogen catalogue # MA5-32593)
(17) E-CADHERIN (Mouse, SCBT catalogue # sc21791, Lot # GR 3360021-2)
(18) E-CADHERIN (Mouse, BD Bio transductions, catalogue # 610182)
(19) Cleaved CASPASE 3 (rabbit, CST catalogue # 9664, Lot # 21)
(20) Phospho HISTONE (Rabbit, CST catalogue # H0412, Lot # 3)
(21) TBR2 (Rrabbit, Abcam catalogue # ab23345, Lot GR3378361-1)
(22) PAX6 (Rabbit, Abcam catalogue # ab195045,Lot GR3339171)
(23) PROX1 (Rabbit, 1:500 Millipore catalogue # ab5475, Lot # 051001268)
(24) β-III TUBULIN (Mouse, Promega catalogue # G7128)
(25) BLBP (Rabbit, Sigma catalogue # ABN14, Lot # 3539833)
(26) ACTIN (Mouse, Sigma catalogue # A2228)
(27) LAMININ (Rabbit, Sigma catalogue # L9393)
(28) β-CATENIN (Rabbit, CST catalogue # 8814, Lot # 5)

Secondary antibodies :
(1) Goat anti-Rabbit Alexa fluor 488 (Invitrogen catalogue #A11034)
(2) Goat anti-Mouse Alexa fluor 594 (Invitrogen catalogue #R37121)
(3) Goat anti-Rabbit Alexa fluor 568 (Invitrogen catalogue #A11011)
(4) Donkey anti-Goat Alexa fluor 568 (Invitrogen catalogue #A32814)
(5) Donkey anti-Mouse IgG Alexa fluor-plus 488 (Invitrogen catalogue #A32766)
(6) Donkey anti-Rabbit IgG Alexa fluor-plus 647 (Invitrogen catalogue #A32733)
(7) Donkey anti-Rabbit IgG 488 (Invitrogen catalogue #A32790)

Validation

All antibodies used in this study were validated previously and the references are attached.

Primary antibodies :

(1) LEF1 (Rabbit, CST catalogue #C12A5),
Link: https://www.cellsignal.com/products/primary-antibodies/lef1-c12a5-rabbit-mab/2230
Citation: Jamieson C, Sharma M, Henderson BR. Regulation of β-catenin nuclear dynamics by GSK-3β involves a LEF-1 positive feedback loop. Traffic. 2011 Aug;12(8):983-99. doi: 10.1111/j.1600-0854.2011.01207.x. Epub 2011 May 13. PMID: 21496192

(2) β-CATENIN (Mouse, BDbiosciences catalogue #610153),
Link: https://www.bdbiosciences.com/us/applications/research/stem-cell-research/cancer-research/human/purified-mouse-anti--catenin-14beta-catenin/p/610153

Citation: Persad S, Troussard AA, McPhee TR, Mulholland DJ, Dedhar S. Tumor suppressor PTEN inhibits nuclear accumulation of beta-catenin and T cell/lymphoid enhancer factor 1-mediated transcriptional activation. J Cell Biol. 2001 Jun 11;153(6):1161-74. doi: 10.1083/jcb.153.6.1161. PMID: 11402061; PMCID: PMC2192018.

(3) RFP (Rabbit, Abcam catalogue #ab62341)
Link: https://www.abcam.com/rfp-antibody-ab62341.html
Citation: Wilson DH, Jarman EJ, Mellin RP, Wilson ML, Waddell SH, Tsokkou P, Younger NT, Raven A, Bhalla SR, Noll ATR, Olde Damink SW, Schaap FG, Chen P, Bates DO, Banales JM, Dean CH, Henderson DJ, Sansom OJ, Kendall TJ, Boulter L. Non-canonical Wnt signalling regulates scarring in biliary disease via the planar cell polarity receptors. Nat Commun. 2020 Jan 23;11(1):445. doi: 10.1038/s41467-020-14283-3. PMID: 31974352; PMCID: PMC6978415.

(4) FZD1 (Rabbit, catalogue #LS-A4150),
Link: https://www.lsbio.com/pathplus-antibodies/pathplus-fzd1-antibody-frizzled-1-antibody-n-terminus-ihc-ls-a4150/547
Data sheet: http://www.naturebiotech.com.tw/MBLI%20GPCR%20Brochure.pdf
Specificity: as per manufacturer page, for Human FZD1 / Frizzled 1. BLAST analysis of the peptide immunogen showed no homology with other human proteins.

(5) AXIN2 (Rabbit , Abcam catalogue #ab185821),
Link: https://www.abcam.com/axin-2-antibody-ab185821.html
Database link : https://www.uniprot.org/uniprot/Q9Y2T1

(6) TTR (Rabbit, DAKO catalogue #A0002),
Link: https://www.agilent.com/en/product/specific-proteins/multipurpose-antibodies-for-clinical-chemistry/prealbumin-(transthyretin)-(multipurpose)-76966
Citation: Sakaguchi, H., Kadoshima, T., Soen, M. et al. Generation of functional hippocampal neurons from self-organizing human embryonic stem cell-derived dorsomedial telencephalic tissue. Nat Commun 6, 8896 (2015). https://doi.org/10.1038/ncomms9896

(7) AQP1 (Mouse SCBT catalogue # sc25287),
Link: https://www.scbt.com/p/aqp1-antibody-b-11
Citation: Zhang Q, Fu J, Xue X. Inhibition of the expression of aquaporin 1 by RNA interference in pulmonary epithelial cells and its effects on water transport. Mol Med Rep. 2016 Jan;13(1):281-6. doi: 10.3892/mmr.2015.4519. Epub 2015 Nov 6. PMID: 26549133

(8) OTX2 (Mouse, SCBT catalogue # sc-514195),
Link: https://www.scbt.com/p/otx2-antibody-d-8
Citation: Ballabio C, Anderle M, Gianesello M, Lago C, Miele E, Cardano M, Aiello G, Piazza S, Caron D, Gianno F, Ciolfi A, Pedace L, Mastronuzzi A, Tartaglia M, Locatelli F, Ferretti E, Giangaspero F, Tiberi L. Modeling medulloblastoma in vivo and with human cerebellar organoids. Nat Commun. 2020 Jan 29;11(1):583. doi: 10.1038/s41467-019-13989-3. PMID: 31996670; PMCID: PMC6989674.

(9) OTX2 (Goat, Invitrogen, PA5-39887),
Link : https://www.thermofisher.com/antibody/product/OTX2-Antibody-Polyclonal/PA5-39887

(10) AQP1 (Rabbit, Invitrogen catalogue # MA5-32593),
Link : https://www.thermofisher.com/antibody/product/Aquaporin-1-Antibody-clone-JM10-98-Recombinant-Monoclonal/MA5-32593

(11) β-III TUBULIN (Rabbit, CST catalogue # 5666)
link: https://www.cellsignal.com/products/primary-antibodies/b3-tubulin-d65a4-xp-rabbit-mab/5666
Citation: Tang Y, Xiong S, Yu P, Liu F, Cheng L. Direct Conversion of Mouse Fibroblasts into Neural Stem Cells by Chemical Cocktail Requires Stepwise Activation of Growth Factors and Nup210. Cell Rep. 2018 Jul 31;24(5):1355-1362.e3. doi: 10.1016/j.celrep.2018.06.116. PMID: 30067988.

(12) Mouse monoclonal Beta-Catenin, (M3539, Dako Agilent, Santa Clara, California, SAD).
Link: https://www.agilent.com/en/product/immunohistochemistry/antibodies-controls/primary-antibodies/beta-catenin-(concentrate)-76569#literature
Specificity: According to manufacturer website Anti-beta-catenin, clone β-catenin-1 recognized human β-catenin protein in Western blots of human epithelial A431 cells and mouse β-catenin in blots of mouse fibroblast NIH/3T3 cells. No cross-reactivity with α and γ-catenin was observed (6).

(13) Non-phospho (Active) βCatenin (Rabbit,D13A1, Cell Signaling, Leiden, Netherlands)
Link : https://www.cellsignal.com/products/primary-antibodies/non-phospho-active-b-catenin-ser33-37-thr41-d13a1-rabbit-mab/8814
Citation: Tsukiyama, T., Zou, J., Kim, J. et al. A phospho-switch controls RNF43-mediated degradation of Wnt receptors to suppress tumorigenesis. Nat Commun 11, 4586 (2020). https://doi.org/10.1038/s41467-020-18257-3

(14) OTX2 (MA5-15854, Invitrogen, Carlsbad, California, SAD)
Link: https://www.thermofisher.com/antibody/product/OTX2-Antibody-clone-1H12C4B5-Monoclonal/MA5-15854
Specificity: Antibody specificity was demonstrated by detection of differential basal expression of the target across tissue tested owing to their inherent genetic constitution. Relative expression of OTX2 was observed in Mouse retina in comparison to Mouse Brain, Mouse Eye and Mouse Lung using Anti-OOTX2 Monoclonal Antibody (1H12C4B5) (Product # MA5-15854) in Western Blot.

(15) SOX9 (Rabbit, Abcam catalogue #ab1852)
link: https://www.abcam.com/sox9-antibody-epr14335-ab185230.html
Russell JP, Lim X, Santambrogio A, Yianni V, Kemkem Y, Wang B, Fish M, Haston S, Grabek A, Hallang S, Lodge EJ, Patist AL, Schedl A, Mollard P, Nusse R, Andoniadou CL. Pituitary stem cells produce paracrine WNT signals to control the expansion of their descendant progenitor cells. Elife. 2021 Jan 5;10:e59142. doi: 10.7554/eLife.59142. PMID: 33399538; PMCID: PMC7803373.

(16) AQP1 (rabbit, 1:200,invitrogen catalogue # MA5-32593)
Link: https://www.thermofisher.com/antibody/product/Aquaporin-1-Antibody-clone-JM10-98-Recombinant-Monoclonal/MA5-32593

(17) E-CADHERIN (Mouse, SCBTcatalogue#sc21791).
Link: https://datasheets.scbt.com/sc-21791
Citation: Hurtado-Alvarado, G., et al. 2016. A2A adenosine receptor antagonism reverts the blood-brain barrier dysfunction induced by sleep restriction. PLoS ONE 11: e0167236

(18) E-CADHERIN (mouse,1:100 BD Bio transductions, catalogue # 610182 0)
Link: https://www.bdbiosciences.com/en-in/products/reagents/microscopy-imaging-reagents/immunofluorescence-reagents/purified-mouse-anti-e-cadherin.610182
Citation: Miyoshi K, Shillingford JM, Smith GH, et al. Signal transducer and activator of transcription (Stat) 5 controls the proliferation and differentiation of mammary alveolar epithelium. J Cell Biol. 2001; 155(4):531-542. (Clone-specific: Immunohistochemistr

(19) Cleaved CASPASE 3 (rabbit, 1:200 CST catalogue # 9664)
Link: https://www.cellsignal.com/products/primary-antibodies/cleaved-caspase-3-asp175-5a1e-rabbit-mab/9664
Citation: Sun T, Duan L, Li J, Guo H, Xiong M. Gypenoside XVII protects against spinal cord injury in mice by regulating the microRNA-21-mediated PTEN/AKT/mTOR pathway. Int J Mol Med. 2021 Aug;48(2):146. doi: 10.3892/ijmm.2021.4979. Epub 2021 Jun 16. PMID: 34132355; PMCID: PMC8208621.

(20) Phospho HISTONE (rabbit, 1:200 CST catalogue # H0412)
Link: https://www.cellsignal.com/products/primary-antibodies/phospho-histone-h2a-x-ser139-20e3-rabbit-mab/9718
Citation: Yuan J, Adamski R, Chen J. Focus on histone variant H2AX: to be or not to be. FEBS Lett. 2010 Sep 10;584(17):3717-24. doi: 10.1016/j.febslet.2010.05.021. Epub 2010 May 21. PMID: 20493860; PMCID: PMC3695482.

(21) TBR2 (rabbit, 1:200 Abcam catalogue # ab23345)
Link: https://www.abcam.com/tbr2--eomes-antibody-ab23345.html
Citation: Amadei G, Lau KYC, De Jonghe J, Gantner CW, Sozen B, Chan C, Zhu M, Kyprianou C, Hollfelder F, Zernicka-Goetz M. Inducible Stem-Cell-Derived Embryos Capture Mouse Morphogenetic Events In Vitro. Dev Cell. 2021 Feb 8;56(3):366-382.e9. doi: 10.1016/j.devcel.2020.12.004. Epub 2020 Dec 29. PMID: 33378662; PMCID: PMC7883308.

(22) PAX6 (rabbit, 1:500 Abcam catalogue # ab195045)
Link: https://www.abcam.com/pax6-antibody-epr15858-ab195045.html
Citation: Xu L, Zhang M, Shi L, Yang X, Chen L, Cao N, Lei A, Cao Y. Neural stemness contributes to cell tumorigenicity. Cell Biosci. 2021 Jan 19;11(1):21. doi: 10.1186/s13578-021-00531-6. PMID: 33468253; PMCID: PMC7814647.

(23) PROX1 (rabbit, 1:500 Millipore catalogue # ab5475)
Link: https://www.merckmillipore.com/IN/en/product/Anti-Prox-1-Antibody,MM_NF-AB5475?ReferrerURL=https%3A%2F%2Fwww.google.com%2F&bd=1
Citation: Cho KO, Lybrand ZR, Ito N, Brulet R, Tafacory F, Zhang L, Good L, Ure K, Kernie SG, Birnbaum SG, Scharfman HE, Eisch AJ, Hsieh J. Aberrant hippocampal neurogenesis contributes to epilepsy and associated cognitive decline. Nat Commun. 2015 Mar 26;6:6606. doi: 10.1038/ncomms7606. PMID: 25808087; PMCID: PMC4375780.

(24) β-III TUBULIN (mouse, 1:100 Promega catalogue # G7128)
Link: https://www.promega.in/products/protein-detection/primary-and-secondary-antibodies/anti_betaiii-tubulin-mab/?catNum=G7121
Citation: Lee JM, Shih AY, Murphy TH, Johnson JA. NF-E2-related factor-2 mediates neuroprotection against mitochondrial complex I inhibitors and increased concentrations of intracellular calcium in primary cortical neurons. J Biol Chem. 2003 Sep 26;278(39):37948-56. doi: 10.1074/jbc.M305204200. Epub 2003 Jul 3. PMID: 12842875.

(25) BLBP (rabbit, 1:200 sigma catalogue # ABN14),
Link: https://www.sigmaaldrich.com/IN/en/product/mm/abn14
Citation: ajpai VK, Kerosuo L, Tseropoulos G, Cummings KA, Wang X, Lei P, Liu B, Liu S, Popescu GK, Bronner ME, Andreadis ST. Reprogramming Postnatal Human Epidermal Keratinocytes Toward Functional Neural Crest Fates. Stem Cells. 2017 May;35(5):1402-1415. doi: 10.1002/stem.2583. Epub 2017 Mar 5. PMID: 28142205; PMCID: PMC5543412.

(26) ACTIN (mouse, 1:500 sigma catalogue # A2228)
Link: https://www.sigmaaldrich.com/IN/en/product/sigma/a2228
Citation: Yang Y, Liu L, Li M, Cheng X, Fang M, Zeng Q, Xu Y. The chromatin remodeling protein BRG1 links ELOVL3 trans-activation to prostate cancer metastasis. Biochim Biophys Acta Gene Regul Mech. 2019 Aug;1862(8):834-845. doi: 10.1016/j.bbagrm.2019.05.005. Epub 2019 May 30. PMID: 31154107.

(27) LAMININ (rabbit, 1:200 sigma catalogue # L9393)

Link: https://www.sigmaaldrich.com/IN/en/product/sigma/l9393?gclid=CjwKCAjw7--KBhAMEiwAxfpkWPog08loN-20zjyga5jYdAui7WHSiqA5v0kKj8YYhl7AbA5qQgTbGxoCAhYQAvD_BwE
Citation: Shin Y, Moriya A, Tohnishi Y, Watanabe T, Imamura Y. Basement membrane-like structures containing NTH α1(IV) are formed around the endothelial cell network in a novel in vitro angiogenesis model. Am J Physiol Cell Physiol. 2019 Aug 1;317(2):C314-C325. doi: 10.1152/ajpcell.00353.2018. Epub 2019 Jun 12. PMID: 31188637; PMCID: PMC6732425.

(28) β-CATENIN (rabbit 1:50, CST catalogue # 8814)
Link: https://www.cellsignal.com/products/primary-antibodies/non-phospho-active-b-catenin-ser33-37-thr41-d13a1-rabbit-mab/8814
Citation: oga A, Yagabasan B, Herrmanns K, Godbersen S, Silva PN, Denzler R, Zünd M, Furter M, Schwank G, Sunagawa S, Hardt WD, Stoffel M. miR-802 regulates Paneth cell function and enterocyte differentiation in the mouse small intestine. Nat Commun. 2021 Jun 7;12(1):3339. doi: 10.1038/s41467-021-23298-3. PMID: 34099655; PMCID: PMC8184787.

Secondary antibodies:

(1) Goat Anti Rabbit Alexa fluor 488 (Invitrogen catalogue # A11034)
https://www.thermofisher.com/antibody/product/Goat-anti-Rabbit-IgG-H-L-Highly-Cross-Adsorbed-Secondary-Antibody-Polyclonal/A-11034

(2) Goat Anti mouse Alexa fluor 594 (Invitrogen catalogue # R37121)
https://www.thermofisher.com/antibody/product/Goat-anti-Mouse-IgG-H-L-Cross-Adsorbed-Secondary-Antibody-Polyclonal/R37121

(3) Goat Anti Rabbit Alexa fluor 568 (Invitrogen catalogue # A11011)
https://www.thermofisher.com/antibody/product/Goat-anti-Rabbit-IgG-H-L-Cross-Adsorbed-Secondary-Antibody-Polyclonal/A-11011

(4) Donkey Anti goat Alexa fluor 568 (Invitrogen catalogue # A32814)
https://www.thermofisher.com/antibody/product/Donkey-anti-Goat-IgG-H-L-Highly-Cross-Adsorbed-Secondary-Antibody-Polyclonal/A32814

(5) Donkey anti-mouse IgG Alexa fluor-plus 488 (Invitrogen catalogue # A32766)
https://www.thermofisher.com/antibody/product/Donkey-anti-Mouse-IgG-HL-Highly-Cross-Adsorbed-Secondary-Antibody-Alexa-Fluor-Plus-488/A32766

(6) Donkey anti-rabbit IgG Alexa fluor-plus 647 (Invitrogen catalogue # A32733)
https://www.thermofisher.com/antibody/product/Goat-anti-Rabbit-IgG-H-L-Highly-Cross-Adsorbed-Secondary-Antibody-Polyclonal/A32733

(7) Donkey anti-rabbit IgG 488 (invitrogen catalogue # A32790)
https://www.thermofisher.com/antibody/product/Donkey-anti-Rabbit-IgG-H-L-Highly-Cross-Adsorbed-Secondary-Antibody-Polyclonal/A32790

Eukaryotic cell lines

Policy information about cell lines

Cell line source(s)	The human ES cell line WIBR3, obtained from the Whitehead Institute for Biomedical Research, (https://hpscreg.eu/cell-line/WIBRe001-A)
Authentication	The cell line has not been authenticated.
Mycoplasma contamination	The hES cell line WIBR3 was tested for Mycoplasma every month and no contamination was found.
Commonly misidentified lines (See ICLAC register)	No commonly misidentified cell lines were used.

Animals and other organisms

Policy information about studies involving animals; ARRIVE guidelines recommended for reporting animal research

Laboratory animals	The mouse lines used in this study were kind gifts from Kathy Millen (Center for Integrative Brain Research, Seattle Children's Research Institute; Lmx1aCre line), Raj Awatramani (Department of Neurology and Center for Genetic Medicine, Northwestern University Feinberg Medical School Chicago, Illinois; β-catenin LOF mice, Michael J. Holtzman (University of Washington, St. Louis), USA; Foxj1Cre line. The Ai9 reporter mouse line was obtained from JAX labs Stock No. 007909.<br>All animals were kept in an ambient temperature and humidity, with a 12hr light-dark cycle and food available ad libitum. Noon of the day of the vaginal plug was designated as embryonic day 0.5 (E0.5). Controls were littermates wherever possible. Control

embryos/pups carrying the Ai9 reporter to mark the Lmx1a or Foxj1 lineage carried the respective Cre transgenes. Breeding colonies were maintained with the female breeders carrying the Lmx1aCre and Foxj1Cre transgenes since this germline recombination occurs in the testes of these lines. Primers used for genotyping were: Cre F: 5’ATTTGCCTGCATTACCGGTC3’, Cre R: 5’ATCAACGTTTTCTTTTCGG3’, Cre positive DNA shows a band at 350bp. For detecting β-catenin LOF (Exon2-6 floxed) following primers were used: RM41 5’AAGGTAGAG TGATGAAAGTTGTT3’; RM42 5’CACCATGTCCTCTGTCTATTC3’; RM43 5’TACACTATTGAATCACAGGGACTT3’. The PCR shows 221bp as Wild type band while 324bp as band corresponding to floxed allele. For genotyping β-catenin GOF (Exon 3 floxed) FP 5’ GCTGCGTGGACAATGGCTAC3’ and RP 5’GCTTTTCTGTCCGGCTCCAT3’25 was used which detects conditional allele at 550bp and wild type band at 350bp in PCR.

The age of embryos/ pups are mentioned in the figures.

Wt Swiss mouse were used for Figure 1h-k.

For Lmx1aCre::β-cat GOF and corresponding controls following ages are used in this study. E12.5 (Figure 2a & f, 3a, 6a-c, Supplementary Figure S4 b & c), E13.5 (Figure 3a, 6d-l, Supplementary Figure S4d, S7 a, b & c), E14.5 (Figure 2b, d, e &h, 3b, c, f, e and j, 4a), E15.5 (Figure 3a), E16.5 (Figure 4d, Supplementary Figure S5a & b), E18.5 (Figure 4f & g), P0 (Supplementary Figure S2f, S3d).

For Lmx1aCre::β-cat LOF and corresponding controls following ages are used in this study. E12.5 (Supplementary figure S2a, S3c), E16.5 (Supplementary Figure S2c, e & g), P0 (Supplementary Figure S2f, S3d).

For Foxj1Cre::β-cat GOF and corresponding controls following ages are used in this study. E 10 (Figure 5b), E12.5 (Figure 5b), E16.5 (Figure 5c & f, Supplementary Figure S6 a-c).

Wild animals	None
Field-collected samples	None
Ethics oversight	All animal protocols were approved by the Institutional Animal Ethics Committee of the Tata Institute of Fundamental Research, Mumbai, India.

Note that full information on the approval of the study protocol must also be provided in the manuscript.

Human research participants

Policy information about studies involving human research participants

Population characteristics	GW11 and 13 fetal brains were analyzed in this study. The fetal age was estimated based on crown-rump length (CRL, in mm), pregnancy records, and histological findings and expressed as GW. In addition, the correlation of maturational parameters (CRL, body mass, pregnancy records, and sonographic examination) revealed no evidence of growth retardation or malformations.
Recruitment	Not applicable. There is no potential self-selection bias or other biases that are likely to impact the results. The selection of tissue samples was based on the post-mortem delay, consistency and quality of the tissue for histological procedures, and developmental parameters.
Ethics oversight	The Institutional Ethical Review Board of the School of Medicine, University of Zagreb, and University Hospital Center Zagreb has approved the procedure for collecting postmortem brain samples (UHC Zagreb EP02/21AG; UZSM: 641-01/19-02/01).

Note that full information on the approval of the study protocol must also be provided in the manuscript.
